# Conformational equilibrium in supramolecular chemistry: Dibutyltriuret case

**DOI:** 10.3762/bjoc.11.227

**Published:** 2015-11-05

**Authors:** Karina Mroczyńska, Małgorzata Kaczorowska, Erkki Kolehmainen, Ireneusz Grubecki, Marek Pietrzak, Borys Ośmiałowski

**Affiliations:** 1 Faculty of Chemical Technology and Engineering, UTP University of Science and Technology, Seminaryjna 3, PL-85326 Bydgoszcz, Poland; 2 Department of Chemistry, University of Jyväskylä, P.O. Box 35, FI-40014, Jyväskylä, Finland

**Keywords:** association, hydrogen bonding, NMR, rotamerism, supramolecular chemistry

## Abstract

The association of substituted benzoates and naphthyridine dianions was used to study the complexation of dibutyltriuret. The title molecule is the simplest molecule able to form two intramolecular hydrogen bonds. The naphthyridine salt was used to break two intramolecular hydrogen bonds at a time while with the use of substituted benzoates the systematic approach to study association was achieved. Both, titrations and variable temperature measurements shed the light on the importance of conformational equilibrium and its influence on association in solution. Moreover, the associates were observed by mass spectrometry. The DFT-based computations for complexes and single bond rotational barriers supports experimental data and helps understanding the properties of multiply hydrogen bonded complexes.

## Introduction

The hydrogen bond (HB) is one of the most common non-covalent interactions. Since it stabilizes, for example, the double helix of DNA and influences peptide folding it is quite reasonable to assume that HBing is crucial for existence of life. It is also present in many small molecules acting as an intramolecular configurational lock. This is realized in hydrazones [1], heterocyclic urea derivatives [2], molecules exhibiting photoexcited proton transfer [3] and other compounds [4–6] reported also by us [7–10]. The intramolecular HBing present in some heterocycles results in a molecular geometry suitable for association by multiple hydrogen bonding [11–23] making possible the formation of, inter alia, stable supramolecular polymers [24–30]. Such polymerization needs properly prearranged monomers with intermolecular hydrogen bonding patterns fitting between molecules. In this sense the conformational freedom is a main limiting factor in molecular design. On the other hand in forms stabilized by an intramolecular hydrogen bond it is possible to break an intramolecular HB and formation of a complex in alternative rotameric state. This observation gives the opportunity to control such processes [2] making conformational equilibrium [4,31–35] one of the factors, or a tool, that should be taken into account in molecular design. To the best of our knowledge there are only few publications focused on simple molecules capable to form two intramolecular HBs [36–37] that break upon association. This phenomenon is still under discussion [37–39]. The said HB breakage and a conformational change may only be realized via strong enough interaction between host (**H**) and guest (**G**) molecules. This is because, as Etter's rules [40] state, intramolecular hydrogen bonding is stronger than intermolecular one and more probable due to the entropy reasons.

In order to construct a molecule capable to form intramolecular hydrogen bonds one should bear in mind some conditions: a) such a molecule must contain a hydrogen bond donor and acceptor in a close proximity and this is especially true when one assumes that b) a six-membered quasi-ring stabilized by hydrogen bonds is preferred over the five-membered one and that c) from the supramolecular/intermolecular interactions point of view the most efficient association exists in complexes in which all hydrogen bond donors belong to one molecule and all hydrogen bond acceptors to the other. The last condition was tested for quadruple hydrogen bonded associates [14], while the basis of this phenomenon are secondary interactions [41] that act diagonally between neighbouring hydrogen bonding sites.

The breakage of single intramolecular hydrogen bonding upon association leading to rotamerism was our motivation to search for more complex systems than previously reported [2,9–10]. Since the amide group is common in biomolecules we have focused on dibutyltriuret (**1**) that contains NH and CO groups. This molecule fulfils the needed properties (a–c above). The parent triuret forms two intramolecular hydrogen bonds in the solid state [42] and upon cooling [43]. The association of parent triuret with cations has already been studied by MS [44] while its interaction preferences with anions in solution are not known. On the other hand the tris-urea derivatives with a spacer between NHCONH groups were used in several supramolecular complexes including those with encapsulated anions [45–46], sensing nerve agents [47] or in self-healing materials [48]. In triuret derivatives no such spacer is present yielding a DDDD (D – hydrogen bonding donor) pattern in its linear form. The synthesis of dibutyltriuret was previously described [49] but we used an alternate method (see experimental part). It is worth mentioning that triuret is known as a byproduct of the uric acid degradation [44].

**Figure 1:**
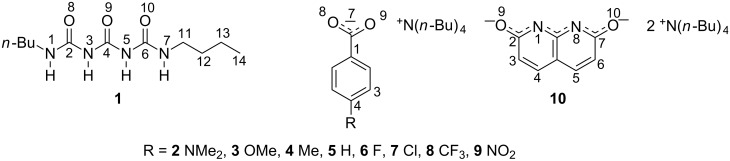
The compounds used in this study and their atom numbering.

The goal of this study is to probe the subjected molecule by anionic counterparts in order to obtain its interaction scheme and to study its intra- vs intermolecular HBing. For that purpose the anionic counterparts chosen are 4-substituted benzoates **2**–**9** and ditetrabutylammonium 1,8-naphthyridin-2,7-diolate (**10**) (Figure 1).

The benzoate anions forming two hydrogen bonds [10] were chosen in order to obtain a series of anions with tuneable HB acceptor properties, while the naphthyridine derivative was used to test if the dibutyltriuret is able to exist in linear form without any intramolecular HBs and stabilized by four intermolecular ones.

**Figure 2:**
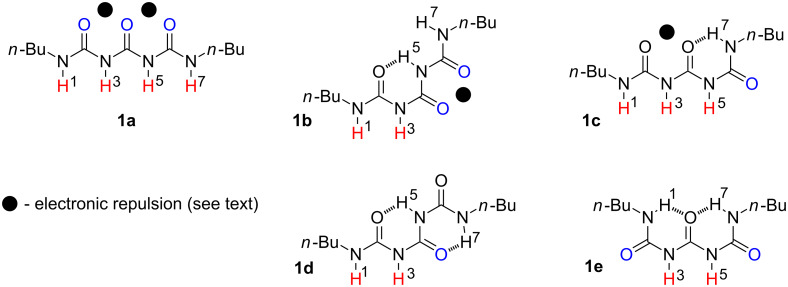
Possible conformations of **1**.

In general the dibutyltriuret molecule carrying four hydrogen bond donors (D, red, Figure 2) and three hydrogen bond acceptors (A, blue) can exist in various conformations stabilized by one or two intramolecular hydrogen bonds and destabilized by electronic repulsions (black dot) in some of them [50].

**Figure 3:**
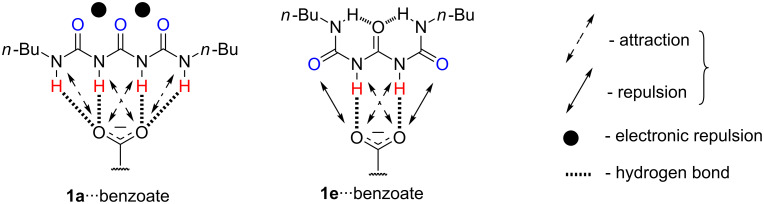
Driving forces influencing association exemplified on two "extreme" conformations of **1**∙∙∙benzoate.

The conformations of **1** correspond to the following hydrogen-bonding patterns: **1a** DDDD, **1b** DDA, **1c** DDDA, **1d** DDA, and **1e** ADDA. This means that the subjected compound may associate by quadruple (in **1a**, **1c** and **1e**), triple (in **1b** and **1d**) and double (all forms) hydrogen bonding with suitable counterparts. For example, DDDD pattern in **1a** should be able to interact with AAAA of **10**. On the other hand all conformations can associate with **2**–**9** by interaction with a DD part of listed patterns. The principal interactions in **1** (Figure 2) or its complex (Figure 3) are: a) multiple hydrogen bonding, b) secondary attractive or repulsive interactions and c) electronic repulsions.

As reported recently by us the intermolecular electronic repulsions [51] can be crucial because they influence on the selectivity of association or the relative population of rotamers [50]. In the current study these forces coexist with other ones in the same molecule.

## Results and Discussion

### Studies by NMR techniques

#### Properties of **1**

**Figure 4:**
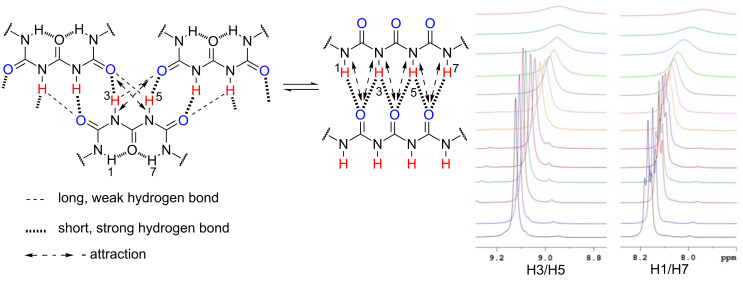
Two possible, extreme multiple hydrogen bonded multimeric structures of **1** and VT ^1^H NMR spectra (from +25 to −40 °C, low temperatures at bottom, CDCl_3_).

The dilution studies for **1** provided its self-association constant *K*_self_ = 170 M^−1^ (CDCl_3_, rt, Supporting Information File 1, Figure S1). The variable temperature (VT) ^1^H NMR spectra reveal that NH protons are quite inert for the temperature change. The broad singlet observed at 8.95 ppm (rt) shifts to 9.12 ppm upon cooling the sample (−40 °C) while the signal observed at 7.89 ppm (rt) shifts to 8.17 ppm (−40 °C) splitting into a sharp triplet at −15 °C revealing the signal originates from H1/H7 with ^3^*J*_(H,H)_ = 5.42 Hz (–CON*H*-C*H*_2_–). The said sharpening is related to the loss of the molecule’s flexibility at lowered temperatures caused by intramolecular hydrogen bonding. At the same time the intermolecular hydrogen bonding is bifurcated in the (di)multimer of **1** (Figure 4).

That definitely also influences its chemical shift. It is worth keeping in mind that together with temperature lowering two events coexist – limited dissociation of associate and enhancement of stabilization of forms locked by intramolecular hydrogen bonding making forms **1e** or **1d** the major ones. Since the spectrum recorded at low temperature shows the compound is symmetric we concluded that the major form is **1e**.

On the other hand **1a** is another extreme form that should be taken into account. The self-associated **1a** must fulfil two prerequisites to exist: a) breakage of the intramolecular hydrogen bond(s) and b) association of two (or more) molecules of **1a** that are much less rigid structures than other ones locked by intramolecular HB. The probability of existence of form **1a** is low at lowered temperatures. The signal broadening at room temperature is caused by fast in NMR time-scale equilibrium between various forms. The NOE experiments for **1** (and associated **1**) at lowered temperatures did not gave any unequivocal data regarding the shift of equilibrium towards any form. This excludes the existence of the dimer or multimer of **1a** as the major form at low temperature.

The correlation of chemical shift of H1/H7 and H3/H5 with temperature is high in the range of −40 °C to ca. +5 °C. The chemical shifts deviate from linear function above +5 °C (Supporting Information File 1, Figure S2). Note that H3/H5 behave irregularly at higher temperatures while H1/H7 data fits well to another linear function. This proves that at rt compound **1** exists as a mixture of forms being under dynamic equilibrium. Protons H1/H7 behave linearly, most probably, due to breaking intramolecular hydrogen bonds and formation of intermolecular ones. It is worth mentioning that the negative slope of said function at higher temperatures is more than three times higher than that for lower temperatures. Lower slope for lower temperatures is caused by intramolecular hydrogen bonding making H1/H7 proton not so sensitive in **1e** to temperature change.

#### Association of **1** with benzoates **2–9**

Since **1** exists in a dynamic equilibrium at rt it was reasonable to associate **1** with anionic counterparts with their properties tuned systematically to the substituent effect. The association constants (*K*_assoc_) are collected in Table 1 (see Supporting Information File 1 for figures). Usually the NH/OH protons are used to find *K*_assoc_ but in some cases CH chemical shifts are also useful [9,52]. Here the protons of methylene attached directly to nitrogen atom were observed during the experiments (Supporting Information File 1, Figure S13).

**Table 1:** Association constants of **1**^a^ [M^−1^] measured with the use of H1, H3.

Counterpart (R)	*K*_assoc_^H1^	*K*_assoc_^H3^

**2** (NMe_2_)	110	170
**3** (OMe)	100	160
**4** (Me)	200	180
**5** (H)	280	270
**6** (F)	170	120
**7** (Cl)	190	160
**8** (CF_3_)	160	220
**9** (NO_2_)	210	250

^a^[**1**] = 18.9 mmol dm^−3^, [**2**–**9**] = 10–12 × [**1**], *T* = 293 K, solvent CDCl_3_.

**Figure 5:**
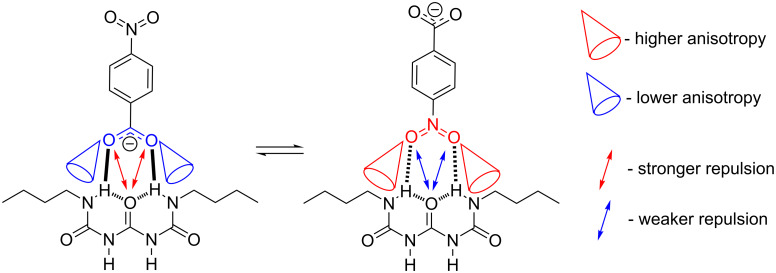
The proposed structure explaining unusual behavior of the titration curve for **1**∙∙∙**9** titration and anisotropy influence on methylene chemical shift.

Changes in the chemical shift of methylene are small but still noticeable. Small changes may be explained by the fact that only one of these protons falls in the anisotropic cone of the associated counterpart (exemplified in Figure 5). Still it was possible to find the complexation-induced shift (CIS) reliably for H11, which is, for example, equal to 0.14 ppm in the **1**∙∙∙**6** complex. It is worth mentioning that for the **1**∙∙∙**9** titration (Supporting Information File 1, Figure S10) an unusual behavior was noticed. The chemical shift of H11 decreases (as in remaining benzoates) at the beginning of the titration and then increases. This may be explained by the alternative binding of **9** with **1e** form as shown in Figure 5.

The following may be concluded based on unnatural behavior of the titration curve: a) within hydrogen-bonded forms present in solution for at least one magnetic anisotropy is important to chemical shift, b) two alternate hydrogen bonded complexes stabilized by CO^−^···HN and NO···HN bridges may be present in **1**∙∙∙**9**, c) this arrangement is observed only for anion carrying another hydrogen bonding group as NO_2_. The dual character of the NO_2_ group (electron-accepting and hydrogen bonding) causes unusual H11 behavior evident (Supporting Information File 1, Figure S10). This type of anisotropic influence on CH_2_ chemical shift may only exist in **1e** form (shown in Figure 5). Only in this form two interacting molecules are in such an arrangement as to locate CH_2_ protons close to the anisotropic cone of the respective moieties in benzoate – no such effect is possible in other form∙∙∙benzoate complexes. This suggests that said alternate **1e**∙∙∙benzoate binding may be present and that NO_2_ group has higher anisotropic influence on CH_2_ shift than -CO_2_^–^ one.

#### Association of **1** with naphthyridine dianion **10**

The *K*_assoc_ equal to 300 M^−1^ for **1**∙∙∙**10** complex (rt) was found based on aromatic doublets in **10**. Although up to four hydrogen bonds stabilize the **1**∙∙∙**10** complex this association is not high due to conformational equilibria and additional stabilization of competitive forms by intramolecular HBing. It was impossible to calculate the association based on NH chemical shifts because NH protons in **1** are not observed at room temperature during titration when the [**G**]:[**H**] (**G** – guest, **H** – host **1**) is higher than 0.2. This, again, suggests fast in NMR time-scale equilibrium. In the **1**∙∙∙**10** complex the titration curve has also a non-standard shape (sigmoidal, see Supporting Information File 1, Figure S11 (inset)).

It is fair to mention that the association constant is loaded with an error higher than in our previous publications and should be treated as an approximate value. This is due to the fact that the titration curves were fitted to have the smallest residuals starting from ca. 0.8 [**G**]:[**H**] ratio till infinite guest concentration. Due to the sigmoidal behavior of all titration curves the fitting is not possible for the beginning of the dataset. Also it is not possible to divide these data into two separate sets as before [9] because no saddle point that could be used for that purposes is present. Still this proves that **1** is in rotational equilibrium, which is dependent from interaction with other molecules.

It is also worth mentioning that for the titration curves for [**G**]:[**H**] = 0 at the curve's inflection point, most probably, two (or more) separate curves overlap. One of these exhibits a decrease of the chemical shift in the beginning of titration with relatively small CIS value and reaches its plateau relatively fast. The said decrease of the chemical shift may only be caused by the rotamerism and change of intra- to intermolecular hydrogen bonding.

#### Substituent effect on association

Recently we have observed the correlation of the *K*_assoc_ and substituent constant in supramolecular complexes [10]. The lack of such correlation for complex of **1** with benzoates may be explained by a) multiple equilibrium (rotamerism in **1**) and b) opposite effects of the substituent on complexes stability (further discussion in Supporting Information File 1).

**Figure 6:**
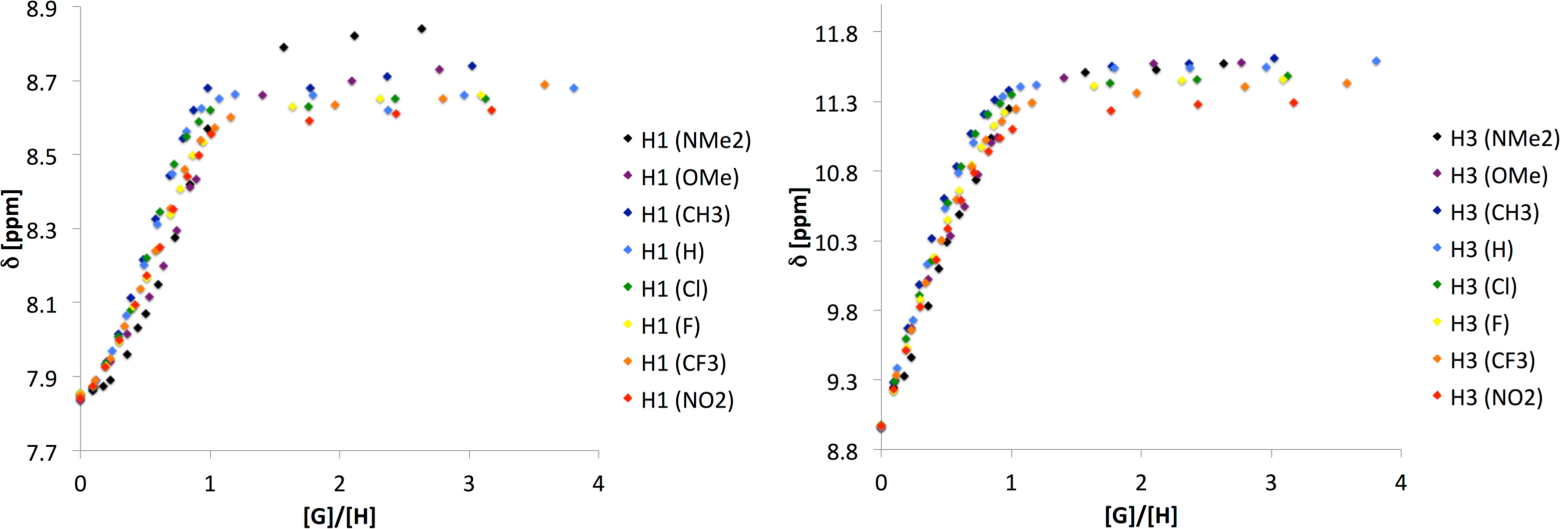
Collective titration curves (H1/H7 and H3/H5 chemical shifts, CDCl_3_) for complex of **1** with substituted benzoates.

The use of substituted benzoate salts gave a set of points shown in Figure 6.

It is easy to see that the substituent effect on chemical shift is not as high as before (compare difference in CIS values in *N*-pyridin-2-ylurea derivative [10]). It is mainly expressed as steeper course of titration curve in case of electron donating substituents than that in case of electron accepting ones. The variable CIS values are directly seen in Figure 6. For a direct comparison between two extreme substituents see Supporting Information File 1, Figure S12.

The higher sigmoidal character is clearly observed for H1/H7, while for H3/H5 the curve is linear-like in the [**G**]:[**H**] range where the H1/H7 function changes from convex to concave. This is caused by different character of H1/H7 vs H3/H5 protons. The steepest titration curve was obtained for **2** (R = NMe_2_, black markers in Figure 6), while for **9** (NO_2_ salt) the curve's shape resemble straight line in the beginning of titration (red markers in Figure 6). The fact that the curve at its beginning is not falling down, as before [9], suggests the association takes place between forms/rotamers involved in intra- and intermolecular hydrogen bonding of similar strength. Probably the *K*_assoc_ is an order of magnitude higher for the more stable complex and this corresponds to the titration data from ca. 0.6–0.7 [**2**–**9**]:[**1**] ratio to infinity. That is understandable since **1e** may form doubly hydrogen-bonded associate at low [**G**]:[**H**] where a competition between **1e**_2_ stabilized by intramolecular hydrogen bonds and its heterocomplex takes place, while at higher [**G**]:[**H**] ratios the probability of formation of intermolecular hydrogen bond is higher. The rotamerism and multiple kind of equilibrium in **1** cause that the general interaction scheme is non-specific yielding a non-linear change of the association constant. On the other hand the regular changes of anion properties are expressed by some regular tendency in the partial titration-derived data (discussion in Supporting Information File 1, page S9). The shapes of curves show that there exist two or more associates at a time especially in the region close to the 1:1 molar ratio. At this point the curve passes through the inflection point located at variable [**2**–**9**]:[**1**] ratio. For more detailed discussion on inflection point analysis refer to Supporting Information File 1. Here it is enough to mention that the position of the inflection point is linearly dependent from the substituent (Hammett) constant taken from the publication by Hansch and Taft [53].

To sum up the substituent effect on association it is worth to stress that the goal of this work was to check how the flexibility of the molecule influences the association. In this case a substituent effect is not observed directly (association constants) but still can be seen in the shape of the titration curve, its inflection point position and CIS values.

#### VT measurements for complexes

The VT ^1^H NMR experiments were conducted to have a deeper insight into the nature of the rotameric equilibrium within the complexes. The following salts were chosen (at various [**1**]:[benzoate] ratios [**1**] = 18.9 mmol dm^−3^): a) unsubstituted **5** (R = H), b) carrying electron donor **2** (R = NMe_2_) and c) carrying electron acceptor **8** (R = CF_3_) and **10**. The salt **9** was not taken into account because, most probably, it forms two types of complexes as discussed earlier. For salts **2**, **5** and **8** the following [**1**]:[benzoate] ratios were used: 1:0.5, 1:1 and 1:2, while for [**1**]:[**10**] 1:0.1, 1:0.5 and 1:1. The temperature range was −40 to +25 °C for benzoates and −70 to +20 °C for the naphthyridine derivative.

#### The benzoate case

The 1:1 [**1**]:[**5**] VT ^1^H NMR data shows the linear δ [ppm] = *f* (T) character in case of partial data (the data deviate from linearity at higher temperatures as before for **1** – Supporting Information File 1, Figure S2). In Table 2 the correlation coefficients, linearity range (*l.r.* in °), slope (*a*) and intercept (*b*) of the fitted linear functions are collected.

**Table 2:** The linear fitting of the δ [ppm] = *f* (T) function for benzoates.

Form(s)	*l.r.*^a^ [°C]	*a* (slope)	*b* (intercept)	*R*^b^	δ *ch.*^c^

**1c** low field	−40 – +15	−0.0146	11.97	0.999	down
**1e**	−40 – −5^d^	−0.0130	11.61	0.996	—^e^
**1d**	−40 – −5^f^	−0.0016	11.06	0.996	up
**1c** high field	−40 – +5	−0.0118	9.52	0.999	down
**1e**	−40 – −20	−0.0018	8.14	0.995	—
**1d**	−40 – +10	−0.0027	8.34	0.995	up

^aL^inearity range, ^b^correlation coefficient, ^c^changes of the chemical shift after reaching *l.r.* limit, ^d^signal not seen above −5 °C, ^e^signal disappear at higher temperatures, ^f^at temperature −5 °C the chemical shift suddenly increases (Figure 8) that was interpreted as **1d**+**5**→**other** form∙∙∙**5** isomerization and averaging of peaks. The similar is realized for H1/H7 (high field signal for **1d**).

**Figure 7:**
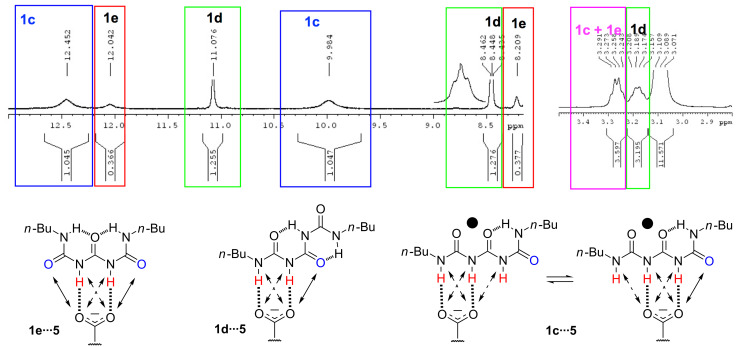
The signal labelling for [**1**]:[**5**] in 1:1 ratio exemplified on the spectra recorded at −40 °C (CDCl_3_) and structure of complexes.

Figure 7 shows the signal labeling. The signals were assigned as follows: a) **1d** by integration (the most populated form – see computational part in Supporting Information File 1), b) **1c** by signal shape (the most broad due to fast “anion shift” between two forms of **1c**∙∙∙**5** within DDD/AA pattern), c) **1e** remaining signal. Some signals are observed at temperatures higher than −40 °C and with various ratios (Figures S17–S25). The comparison of the VT ^1^H NMR spectra with anions carrying various substituents is presented in Supporting Information File 1, Figure S26a–c.

**Figure 8:**
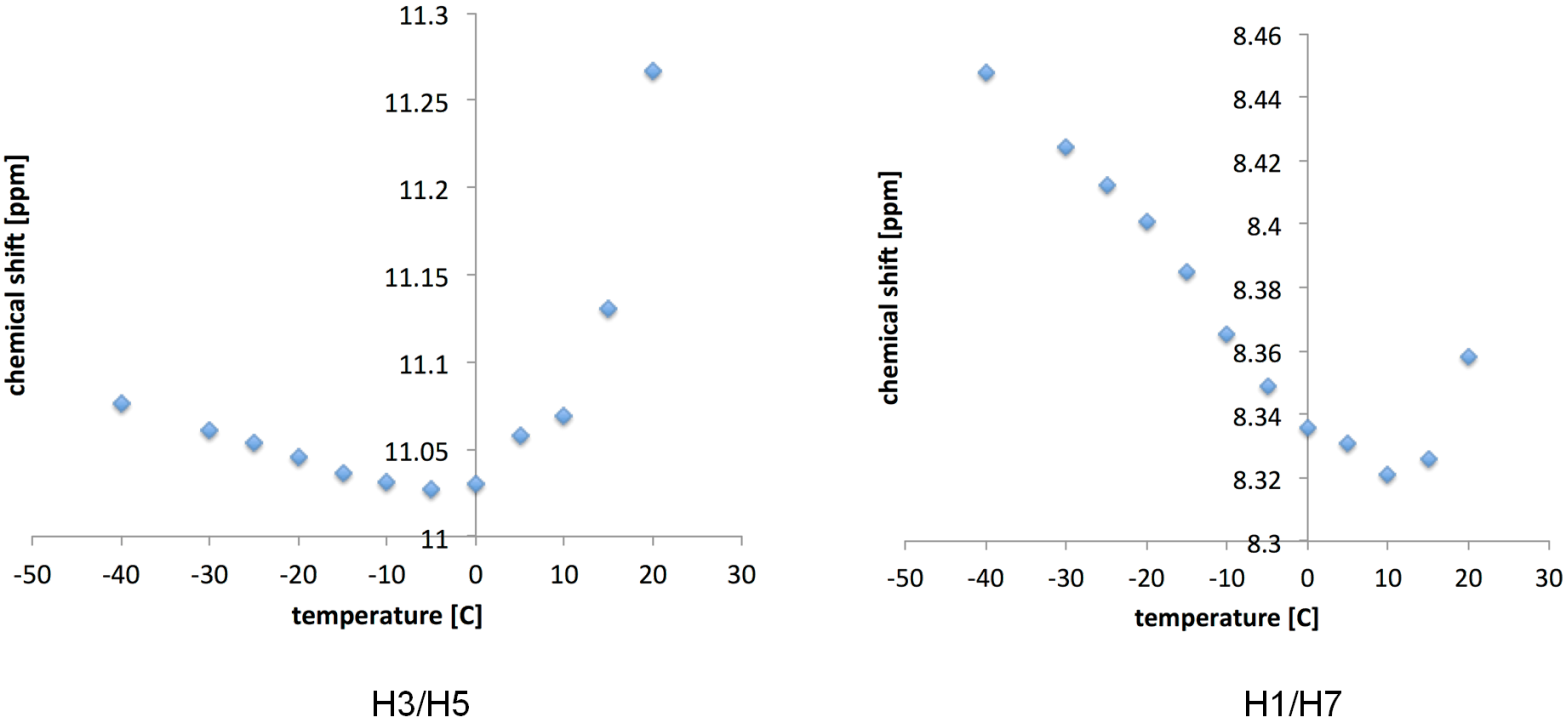
The variable temperature (+20 to −40 °C, CDCl_3_) dependence of the main signals (the highest integral values) in **1d**∙∙∙**5** complex.

The spectrum in Figure 7 shows that at least three forms of **1**∙∙∙**5** can be observed in solution at low temperatures. The signals suggest these forms are symmetric but it is important to keep in mind that some rotational equilibrium may still be present. In **1d** associated to **5** signals of H1/H7 protons are much sharper showing a triplet at 8.45 ppm. The signal of H1/H7 in associated **1e** form at 8.21 ppm lies in the similar region as in **1** (dimer or polymer) at the same temperature and has similar shape (broadened singlet tending to triplet shape), while H3/H5 signals shift from 9.13 (**1**) to 12.04 ppm due to interaction with an anion. The broadest NH signals come from **1c**∙∙∙**5** complex that, most probably, exist in a fast equilibrium as shown in Figure 7. In the aliphatic part of the spectrum, except the methylene of ^+^N(*n-*Bu)_4_ cation (at 3.09 ppm, out of the green box, Figure 7), two characteristic signals are visible. It is worth pointing out that their assignment based on integration is in perfect agreement with the data obtained by integration of NH protons in the same spectrum. The ratio of 3.597/3.195 = 1.126 while the respective sum of NH (H3/H5) integration in **1c** and integration of NH in **1e** divided by integration of NH in **1d** (H3/H5) is 1.411/1.255 = 1.124. Figure 8 shows a rapid change of the chemical shift observed at temperatures ca. 0 to +10 °C for **1d**∙∙∙**5**.

This is caused by fast equilibrium between forms in solution. In this case the signal is averaged and since it was shown in Figure 7 that other than **1d**∙∙∙**5** forms are represented by higher NH chemical shifts the mentioned signal averaging causes increase of the chemical shift and signal broadening at higher temperature (Supporting Information File 1, Figure S21, for example). This may be especially true if one realize that NH groups are involved in intramolecular hydrogen bonding after dissociation of the complex at higher temperatures. Moreover, the proposed forms that are present at low temperatures were calculated and their energetic relations are in agreement with observed data (see data in Supporting Information File 1 and Figure S34 for the energy diagram).

#### The naphthyridine case

**Figure 9:**
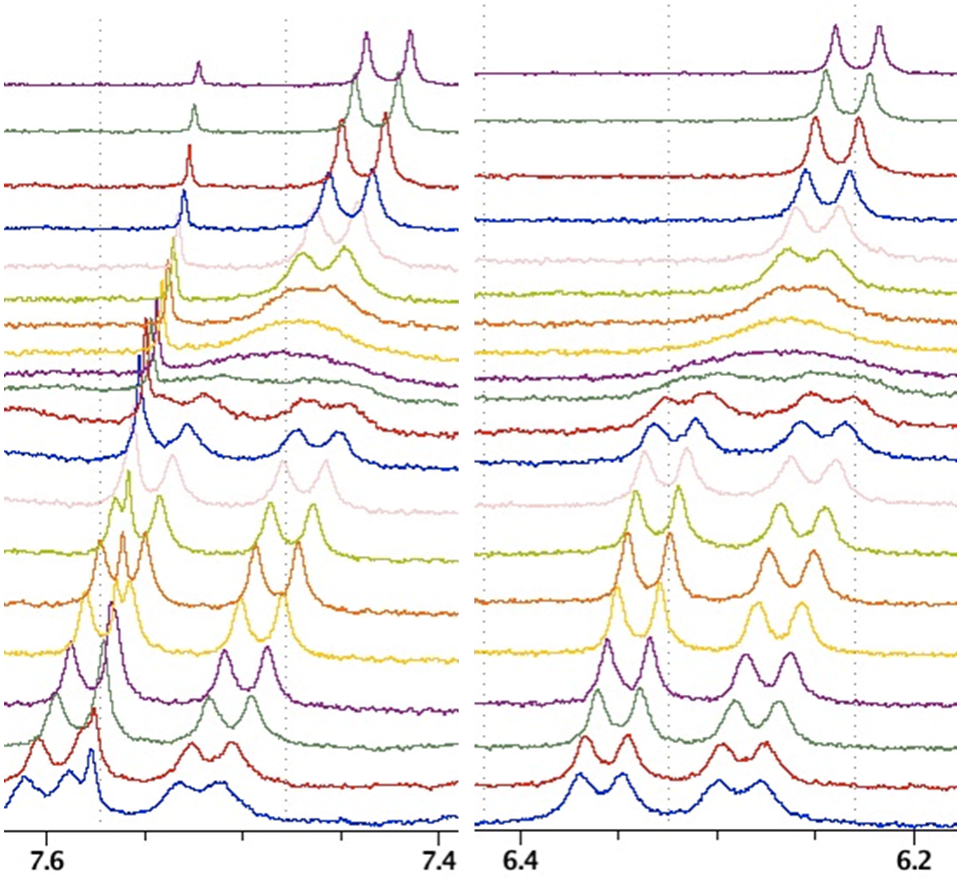
The VT (+20 to −70 °C, CDCl_3_) ^1^H NMR stacked spectra (low temp. at bottom) for **1**∙∙∙**10** in 1:0.1 molar ratio (on the left spectrum a small satellite signal from residual chloroform is seen).

The signal observation difficulties (disappearance of NH peaks) in **1**∙∙∙**10** were overcome by observation of aromatic doublets of **10**. The lack of NH signals in the spectrum may be caused by: a) fast equilibrium or b) the proton transfer between **1** and naphthyridine dianion **10**. While the proton transfer is more probable at higher temperatures and lowering the temperature causes the increase of the population of form with intramolecular hydrogen bond we opt for argument "a". Figure 9 shows the changes in the spectra upon cooling (top spectrum represents +20 °C, spectra were recorded in 5° steps; the last spectrum at bottom represents a temperature of −70 °C).

**Figure 10:**
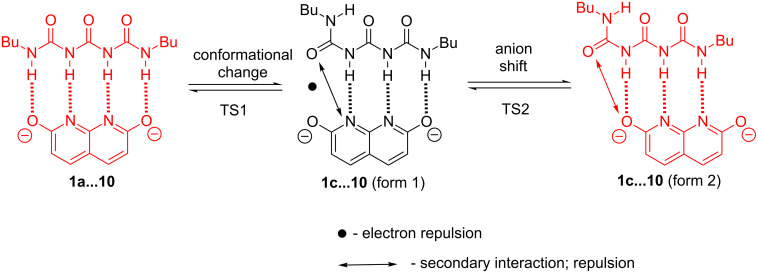
Two most probable forms of **1**∙∙∙**10** complex (in red) with "rotational-shift" path between them.

The coalescence temperature for complex **1**∙∙∙**10** is −15 °C. At temperatures below −25 °C the equilibrium is slow enough to observe an asymmetric complex. It is important to keep in mind that these spectra are recorded for a 1:0.1 molar ratio of **1** and **10** to be sure that most of **10** is associated. From VT ^1^H NMR measurements the Gibb's free energy equal to 66.6 kJ/mol was obtained (Eyring equation). Two forms in **1**∙∙∙**10** complexes are, most probably **1a**∙∙∙**10** (high temperature) and **1c**∙∙∙**10** (low temperature). Between these two rotational transition states exist and one form represented by a local energy minium (Figure 10).

These are transition states related to a) conformational change of **1** and b) shift of the anion **10** along the hydrogen bonding pattern to minimize the electron repulsion. Along the same conformational path another form of **1c**∙∙∙**10** (form 1) is present. This form is, however, less stable than **1c**∙∙∙**10** (form 2) due to electronic repulsion between basic centers in the complex. For detailed discussion that supports experimental findings refer to the *computations* section in Supporting Information File 1.

### Mass spectrometry

To have a more complete view of properties of **1** we applied the combination of soft ionization technique electrospray ionization (ESI) with high resolution mass spectrometry (HRMS) for the study of the association processes of **1** with benzoate **5** and naphthyridine **10** anions and to examine self-association of **1**. The ESI–HRMS was previously successfully used for the study of complexation of triuret derivative with cations [44] and has been demonstrated to provide suitable tool for the structural elucidation of different types of supramolecular complexes, including hydrogen bounded compounds [54–56].

The ESI(−)–HRMS mass spectra of chloroform/methanol solutions containing equimolar concentrations of **1** and benzoate **5**, and of **1** with naphthyridine **10** are shown in Supporting Information File 1 (Figures S36 and S37, respectively). Both mass spectra are dominated by peaks which correspond to: anions of deprotonated **1**; [**1** − H^+^]^−^ (*m*/*z*_calc_ = 257.1613, Figure S36: *m*/*z*_meas_ = 257.16208, Figure S37: *m*/*z*_meas_ = 257.16220 ) and anions formed as a result of self-association and deprotonation of **1**; [**1**_2_ − H^+^]^−^(*m*/*z*_calc_ = 515.3305, Figure S36: *m*/*z*_meas_ = 515.33185; Figure S37: *m*/*z*_meas_ = 515.33213). Less intensive signal observed at ESI(−)–HRMS mass spectrum of **1**∙∙∙**10** (Figure S37) can be assigned to singly charged anions formed via protonation of naphthyridine **10** dianion; [**10** + H^+^]^−^ (*m*/*z*_calc_ = 161.0351, *m*/*z*_meas_ = 161.03475). Protonation of **10** dianion is probably related to the presence of methanol in the solution. Signal noted at *m*/*z* = 419.20560 corresponds to supramolecular complexes formed as a result of association of ions [**1** − H^+^]^−^ and neutral molecules of [**10** + 2H^+^] or alternatively are created via association of neutral molecules of **1** with singly charged ions [**10** + H^+^]^−^. Formation of both types of complexes is possible as both anions [**1** − H^+^]^−^ and [**10** + H^+^]^−^ are present in the solution.

The ESI(+)–HRMS spectrum of **1** dissolved in chloroform/methanol shown in Supporting Information File 1 (Figure S38) confirms that dibutyltriuret also self-associates and forms singly charged cations of monomer [**1** + H^+^]^+^ (*m*/*z*_calc_ = 259.1770, *m*/*z*_meas_ = 259.17675), dimer [**1**_2_ + H^+^]^+^ (*m*/*z*_calc_ = 517.3461, *m*/*z* = 517.34636), and trimer [**1**_3_ + H^+^]^+^ (*m*/*z*_calc_ = 775.5153, *m*/*z*_meas_ = 775.51543) via positive electrospray.

The results of performed ESI–HRMS experiments clearly show that dibutyltriuret can easily self-associate in the chloroform/methanol solution, forming singly charged cations of dimers and trimers in positive electrospray and singly charged anions of dimers in negative electrospray, respectively. Association processes of dibutyltriuret **1** with anionic guests also occur, however presence of methanol in the analytical solutions and/or mechanism of ESI ionization process (in ESI ions are created by the addition or removal of a proton(s)) [57] have non-negligible influence on the elemental compositions and architectures of created supramolecular complexes. Given the high mass accuracy of the HRMS mass spectrometry there can be no question as to the elemental composition or charge of formed ions. However results of performed HRMS experiments do not provide detailed information about the number and location of hydrogen bonds formed between components of created supramolecular species.

## Conclusion

Theoretically the dibutyltriuret studied in this work can exist in five conformations. Most of them can be stabilized by intramolecular hydrogen bonds. VT ^1^H NMR studies reveal that this compound exists with associated benzoate at least in three forms at low temperatures. The dynamic equilibrium causes elimination of regular change of association as a function of substituent constant. However, the substituent change gave a set of data showing its influence on properties of associates. This, in turn, shed light on the conformational state of **1** in the complex. More importantly an interaction with dianion of naphthyridine derivative ascertained that in this complex dibutyltriuret associate by breaking two intramolecular hydrogen bonds. These experimental observations are in line with computations that are very useful in drawing conclusions. The use of benzoates with regularly changed properties may be the method of choice in analysis of complexes. The pre-organization of molecules used in supramolecular chemistry is, in general, important to this field. On the other hand understanding the behaviour of non-rigid molecules may also be useful and a challenging task.

## Experimental

### Synthesis

Compound **1** was obtained by heating urea (1.0 g, 16.7 mmol) and *n*-butyl isocyanate (3.3 g, 33.4 mmol, 1:2 molar ratio) for 24 h under reflux in pyridine (20 mL). Then pyridine was removed under vacuum and the residual was recrystallized three times from EtOH giving 2.67 g of **1** (yield 62%). The tetra-*n*-butylammonium benzoates **2**–**9** and naphthyridine salt **10** were synthesized as described before and were used after keeping for several days in desiccator over P_2_O_5_. Their characterization is given elsewhere while the spectral data for newly synthesized salt **10** and **1** is given below.

### Compound characterization

*N*,*N*'-Bis(*n*-butylcarbamoyl)urea (**1**): Yield 62% (pure compound). ^1^H NMR (CDCl_3_ from TMS) δ 8.98 (bs, 2H), 7.93 (bs, 2H), 3.32 (q, ^3^*J*_H,H_ = 6.3 Hz, 4H), 1.52 (m, 4H,), 1.39 (m, 4H), 0.94 (t, ^3^*J*_H,H_ = 7.3 Hz, 6H); ^13^C (CDCl_3_ from TMS) δ 154.0 (CO), 153.8 (CO), 39.8 (CH_2_), 31.6 (CH_2_), 20.0 (CH_2_), 13.7 (CH_3_); mp 159.8–161.3 °C (EtOH) lit. 160–162 °C [49]; anal. calcd for C_11_H_22_N_4_O_3_: C, 51.15; H, 8.58; N, 21.69; O, 18.58, found: C, 51.04; H, 8.62; N, 21.40.

Di(tetra-*n*-butylammonium) 1,8-naphthyridin-2,7-diolate (**10**): ^1^H NMR (CDCl_3_ from TMS) δ 7.33 (d, ^3^*J*_H,H_ = 8.9 Hz, 2H), 6.07 (d, ^3^*J*_H,H_ = 8.9 Hz, 2H), 3.28 (m, 16H), 1.62 (m, 16H), 1.41 (m, 16H), 0.96 (t, 24H); ^13^C (CDCl_3_ from TMS) δ 167.8 (C), 159.6 (C), 138.3 (CH), 112.4 (CH), 102.4 (C), 58.8 (CH_2_), 24.0 (CH_2_), 19.7 (CH_2_), 13.7 (CH_3_); mp 73.2–75.0 °C; anal. calcd for C_40_H_76_N_4_O_2_: C, 74.48; H, 11.88; N, 8.69; O, 4.96, found: C, 74.29; H, 12.05; N, 8.41.

The NMR titrations and dilution studies were performed as before [58] in CDCl_3_ as a solvent at 20 °C. All spectra were recorded using a Bruker Avance III 400 MHz spectrometer. Mass spectrometry analysis was performed on a Q-Exactive mass spectrometer (Thermo Scientific). The Benesi–Hildebrand equation [59] was used to find association constants while the method proposed by Tan [60] was used for dimerization of **1**. It was assumed that 1:1 stoichiometry is present in all complexes at a high concentration of guest. The only form that could bind with two benzoates is **1a**, but due to the strong repulsion between anions being in close proximity we have excluded this type of associate from detailed considerations. In order to have a further insight into the studied complexes VT ^1^H NMR spectra were recorded in CDCl_3_ dried over molecular sieves. For this purpose three benzoates **2**, **5** and **8** and naphthyridine salt **10** were used. Since some of discussed complexes/rotamers are nonsymmetric the numbering of atoms in **1** is from 1 to 14 although the rotamers **1a** or **1e** shown in Figure 2 are symmetric. Mass spectra were recorded using Q-Exactive mass spectrometer (Thermo Scientific). Data acquisition and analysis were conducted using the Xcalibur (Thermo Scientific) software. All structures were optimized with the use of the Gaussian [61] software with 6-311+G(2d,2p) basis set and PCM [62–63] model of solvation (chloroform). The M05 functional suggested for non-covalent interactions [64–65] was used to sustain the methodology from our previous publications [9–10,50,66]. The Synchronous Transit-Guided Quasi-Newton method [67–68] has been used for optimization of the transition states (rotamerism in **1** and as a complex). In all computations the ZPE (zero-point energy) correction was taken into account. The energy of intermolecular interaction (*E*_int_) was corrected by using BSSE (basis set superposition error) calculations with the counterpoise method [69–70] as implemented in Gaussian with default settings. For all structures the frequency calculations were ran to be sure that the geometry is in an energy minimum. Except for transition states where one imaginary frequency was obtained all other structures gave only real frequencies. The AIM2000 [71] software was used to calculate the properties of the hydrogen bond critical point.

## Supporting Information

File 1 Charts (titrations, function fitting, correlation charts), NMR spectra, optimized geometry data, additional discussion, computational data with detailed comment and mass spectra.
