# Supplementary material for: Conformational equilibrium in supramolecular chemistry: Dibutyltriuret case
Source: Beilstein J Org Chem. 2015 Nov 5;11:2105–16. doi: 10.3762/bjoc.11.227 (PMC4660960; doi:10.3762/bjoc.11.227)
Supplement: File 1 — Charts (titrations, function fitting, correlation charts), NMR spectra, optimized geometry data, additional discussion, computational data with detailed comment and mass spectra. [file Beilstein_J_Org_Chem-11-2105-s001.pdf]

Supporting Information

for

Conformational equilibrium in supramolecular chemistry: Dibutyltriuret case

Karina Mroczyńska[1], Małgorzata Kaczorowska[1], Erkki Kolehmainen[2], Ireneusz Grubecki[1], Marek Pietrzak[1], Borys Ośmiałowski[1,*]

Address: [1]Faculty of Chemical Technology and Engineering, UTP University of Science and Technology, Seminaryjna 3, PL-85326 Bydgoszcz, Poland and [2]Department of Chemistry, University of Jyväskylä, P.O. Box 35, FI-40014 Jyväskylä, Finland

E-mail: Email: Borys Ośmiałowski - borys.osmialowski@utp.edu.pl

*Corresponding author

Charts (titrations, function fitting, correlation charts), NMR spectra, optimized geometry data, additional discussion, computational data with detailed comment and mass spectra

Table of contents

Dilution and titration charts	S2
Collective data for changes in $\delta(CH_2)$ during titrations	S8
Correlation charts	S9
Inflection point analysis	S10
NMR spectra	S12
VT ^{1}H NMR spectra	S14
The NOE spectra	S18
Geometry of the optimized structures	S19
Cartesian coordinates of the optimized structures	S26
Opposite effect of substituent on association (additional discussion)	S42
Additional discussion (HOMA based and substituent effect)	S43
Computations	S46
Mass spectra	S51
References	S53

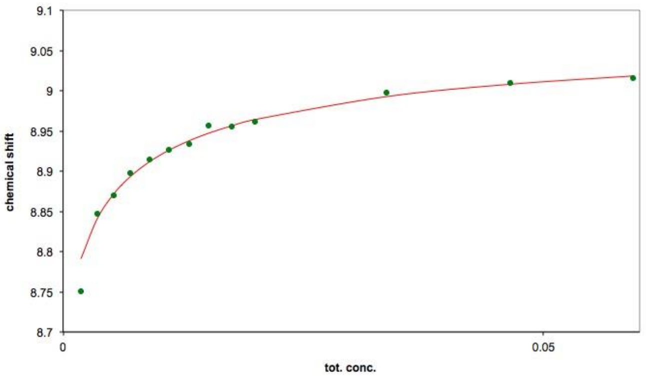

Figure S1. Dilution chart for **1** (r.t., in $CDCl_3$ at r.t., conc. form 1.8 to 60 mmol/dm^3)

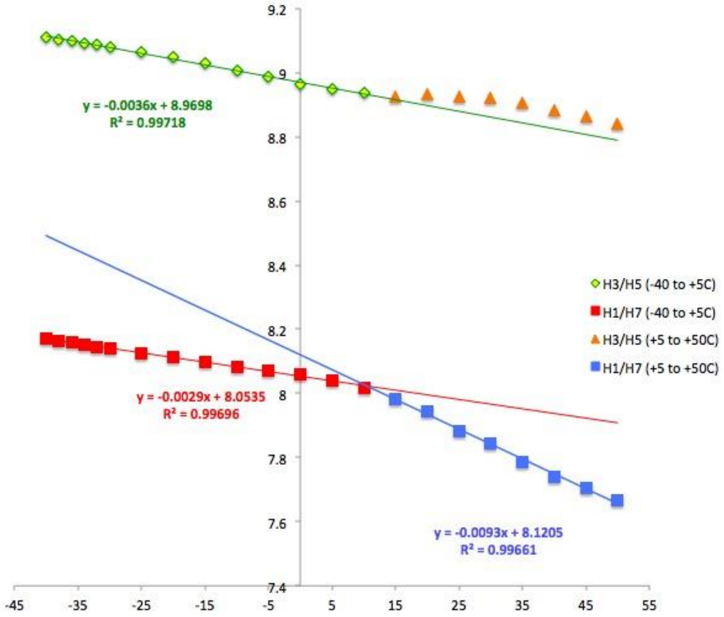

Figure S2. The dependence of the chemical shift from temperature (results of ^{1}H VT NMR experiments for **1,** conc. = 18.9 mmol/dm^3)

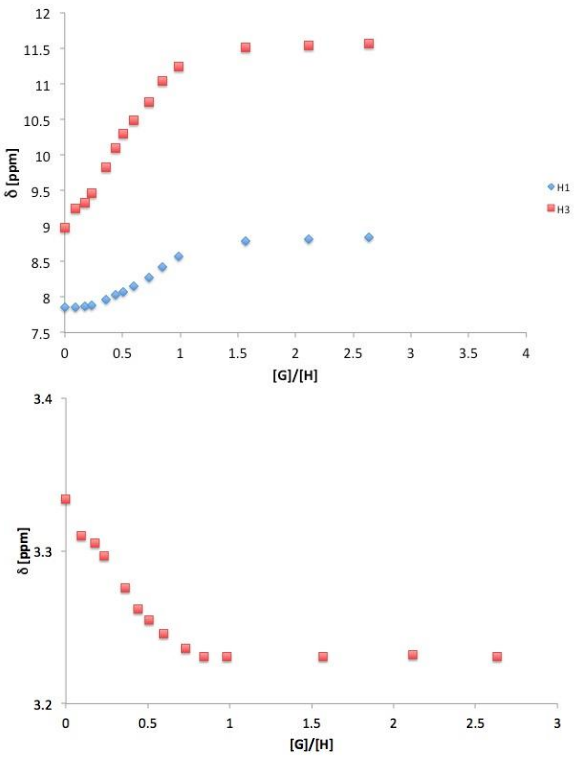

Figure S3. The titration chart for **1···2** (NMe_2, H1 and H3 top, H11 bottom)

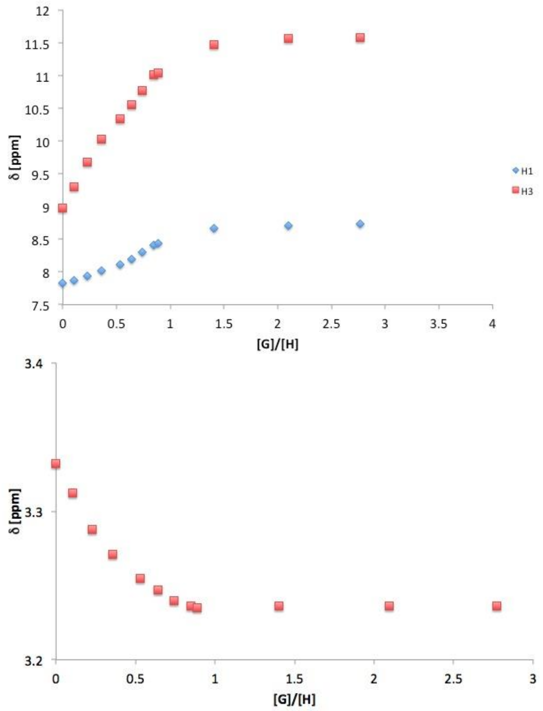

Figure S4. The titration chart for **1···3** (OMe, H1 and H3 top, H11 bottom)

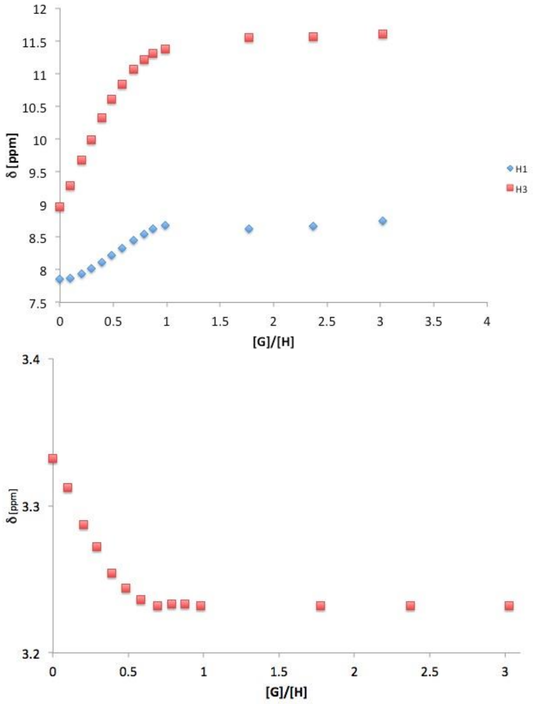

Figure S5. The titration chart for **1···4** (Me, H1 and H3 top, H11 bottom)

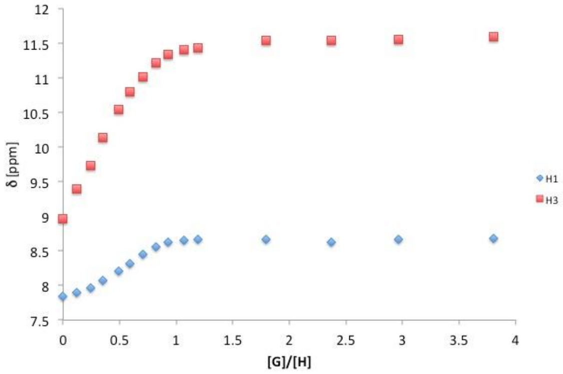

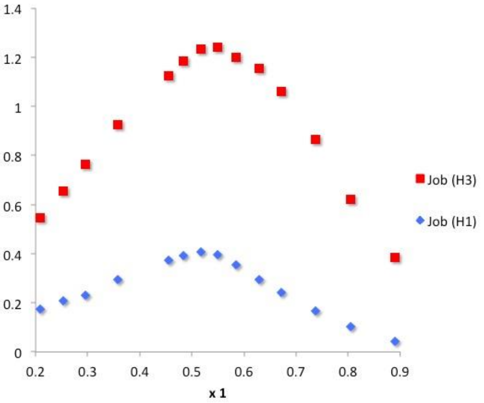

Job's plot.

Comment: For all remaining titration the maximum in Job's plot is located at ca. 0.5 fraction of **1** giving a proof of 1:1 stoichiometry in the complex.

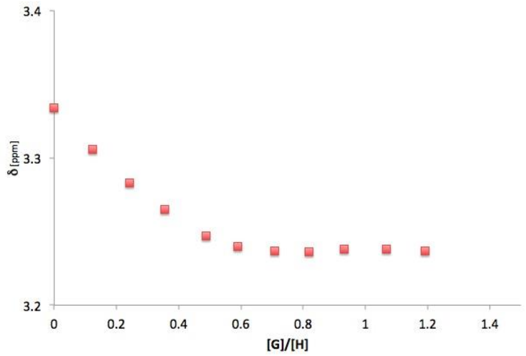

Figure S6. The titration chart for **1···5** (H, H1 and H3 top, H11 bottom)

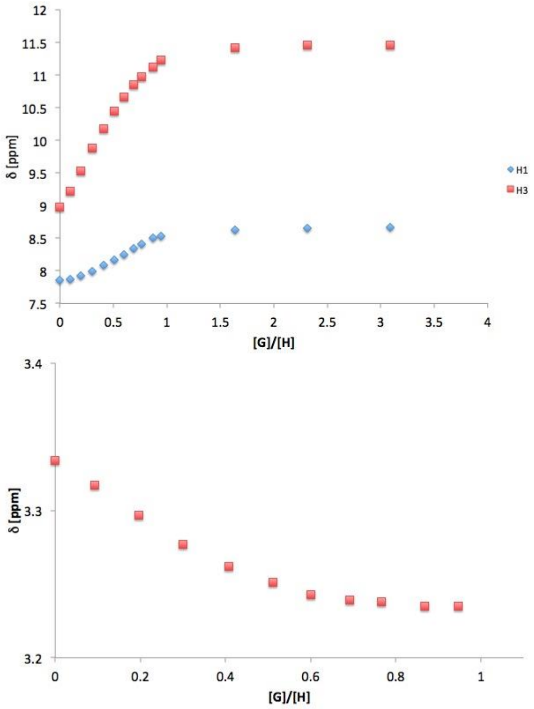

Figure S7. The titration chart for **1···6** (F, H1 and H3 top, H11 bottom)

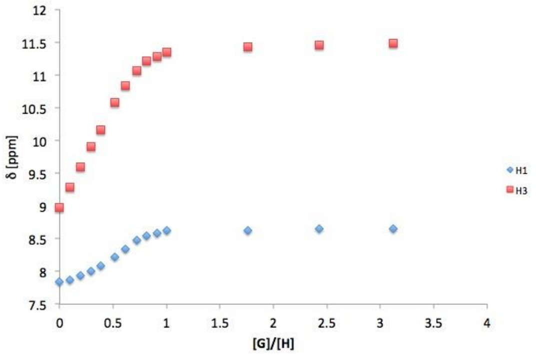

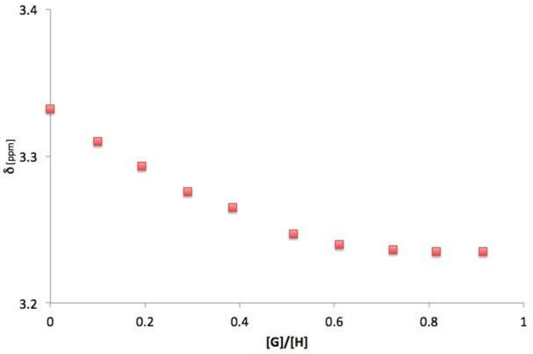

Figure S8. The titration chart for **1···7** (Cl, H1 and H3 top, H11 bottom)

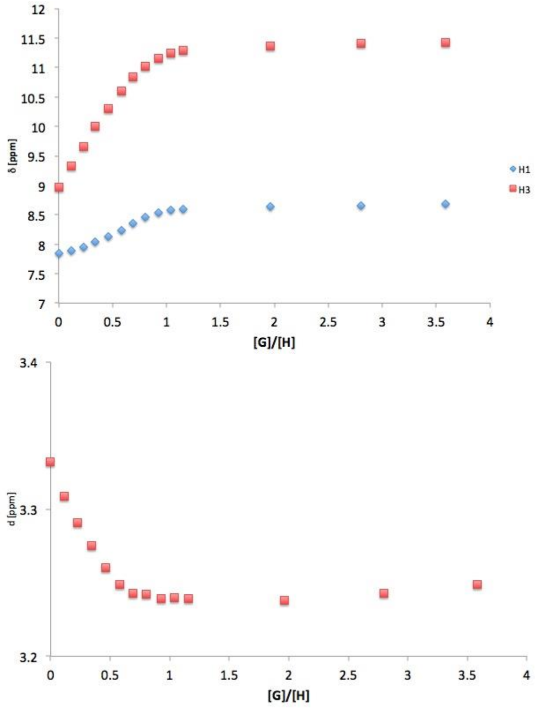

Figure S9. The titration chart for **1···8** (CF_3, H1 and H3 top, H11 bottom)

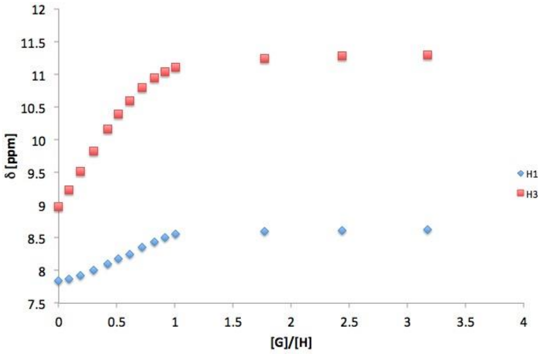

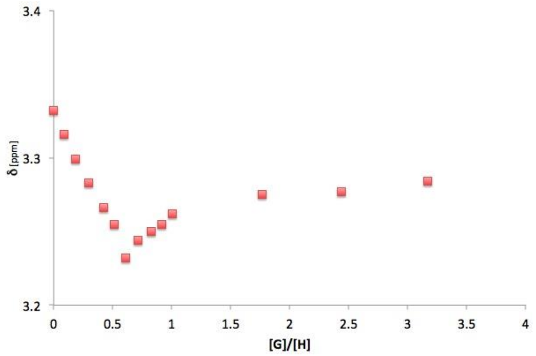

Figure S10. The titration chart for **1···9** (NO$_2$, H1 and H3 top, H11 bottom)

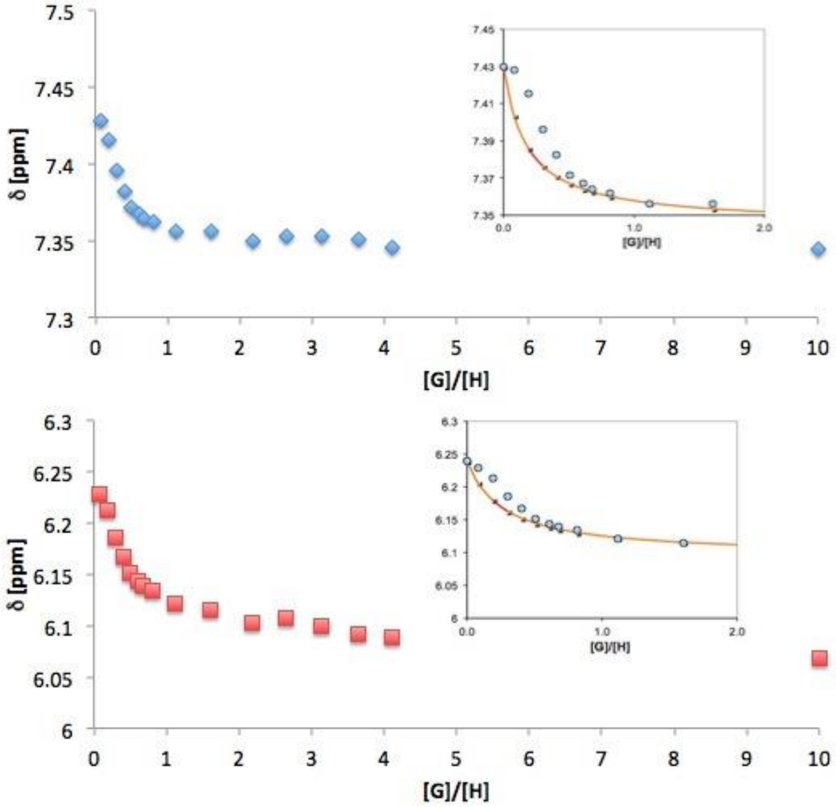

Figure S11. The titration chart for **1···10**

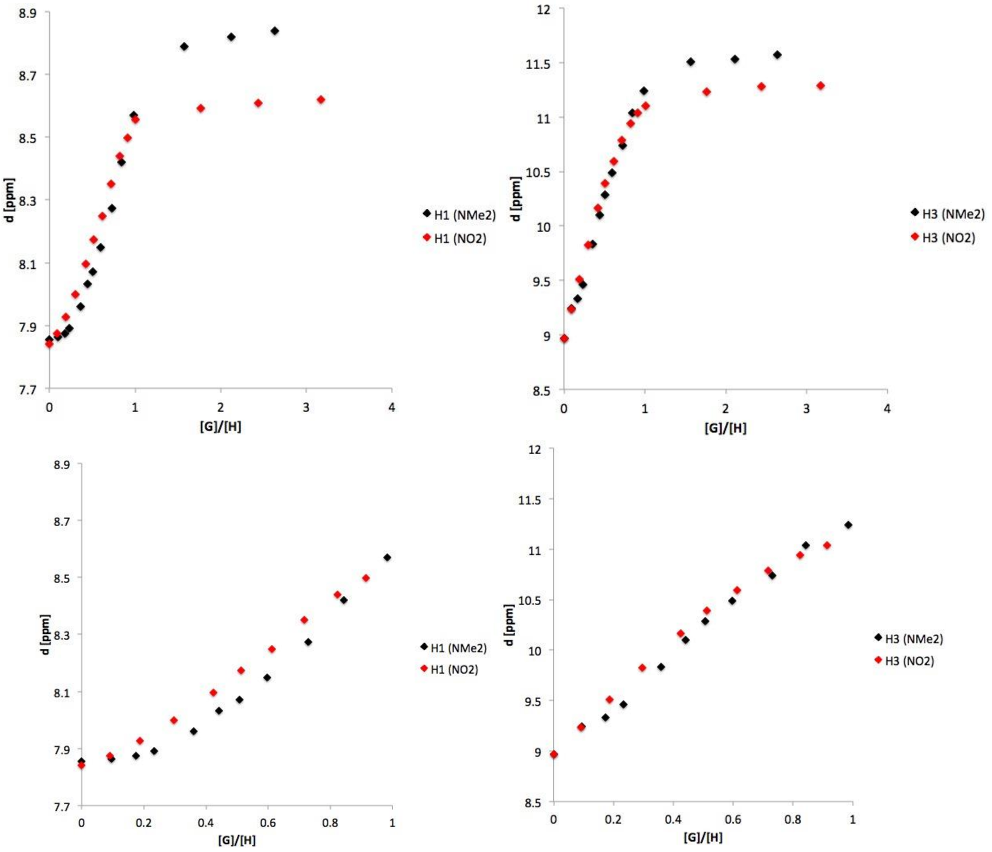

Figure S12. Comparison of titration curves for titrations with **2** and **9** (the full data on top, [**G**]/[**H**] range equal to 0-1 bottom)

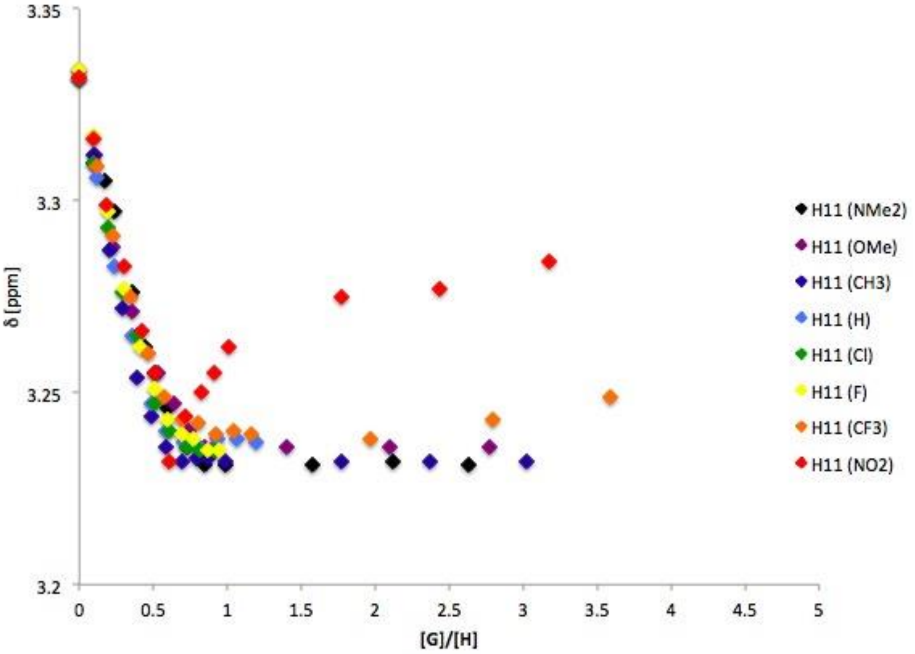

Figure S13. The collective CH_2-based titration chart

The titration curves behave like a linear function in the range between ca. 0.5 and 0.8 [**G**]:[**H**]. For this range the straight line was fitted giving various *slope* values. The said *slope*s are dependent from the substituent constant after removing some points. For the R = NMe_2, Me, Cl, CF_3 and NO_2 set the correlation coefficient is equal to R = 0.94 (Figure S14). This value shows the tendency in the substituent-driven association in the region of fast conformational changes. The deviations of the remaining three substituents from the trend may be caused by a small amount of water in the samples - although we used dried solvents some compounds may be more hygroscopic than others.

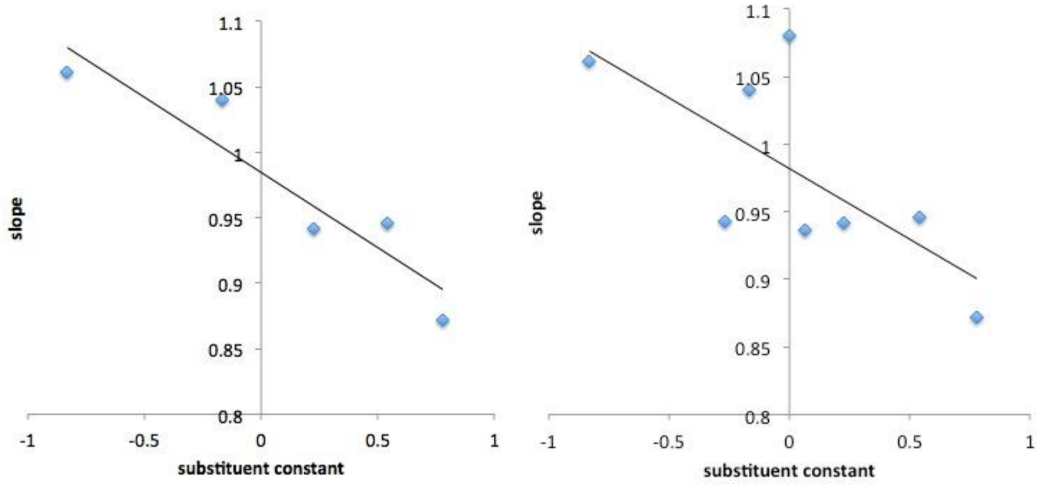

R = 0.94 *R*=0.71

Figure S14. The correlation of the *slope = f (substituent constant)* from partial titration data - selected points (left) and the full set of data (right) in titration charts

Inflection point analysis

Table S1. The results (inflection points) of the fitting the sigmoidal dose-response function to the titration data

		sigmoidal dose-response function			
R	σ	[**G**]/[**H**]^{infl} value for H1/H7	δ^{infl} [ppm] value for H1/H7	[**G**]/[**H**]^{infl} value for H3/H5	δ^{infl} [ppm] value for H3/H5
NMe_2	-0.83	0.851	8.336	0.509	10.200
OMe	-0.27	0.727	8.268	0.467	10.205
Me	-0.17	0.513	8.261	0.322	10.171
H	0	0.449	8.225	0.319	10.142
F	0.06	0.583	8.233	0.373	10.122
Cl	0.23	0.482	8.227	0.330	10.132
CF_3	0.54	0.554	8.235	0.352	10.094
NO_2	0.78	0.539	8.206	0.355	10.037
R^a		*0.726*	*0.898*	*0.675*	*0.925*

^{a} – correlation coefficient

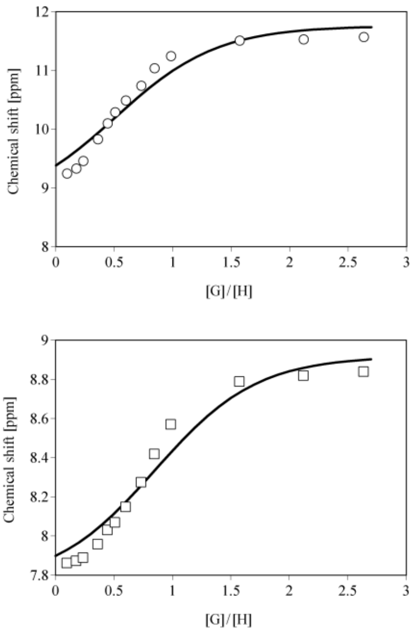

Figure S15. Example of the fitting of the dose-response curve to the experimental data (**1···2** titration) used for finding inflection points. See the main text for description.

Comment: the relatively high residuals in the fitted functions suggest there are several forms of **1** in fast equilibrium.

Since the data of the ^{1}H NMR titration shown that more than two forms are present in solution making the association non-specific the data were treated mathematically to find if the inflection point of all titration curves is depended from the substituent (the dose-response function was used[1]). The position of the inflection point correlates with substituent constant (Table S1). It is important to note that the correlation between position of the inflection point on the Y-axis (δ^{infl}[ppm]) and σ values (for linear function $\delta^{infl}=f(\sigma)$) is higher than that for X-axis ([**G**]:[**H**]^{infl})

data *vs* σ ([**G**]:[**H**]^{infl}=$f(\sigma)$).The lower values of correlation coefficient in case of [**G**]:[**H**]^{infl} *vs* σ may be explained by shift in rotameric equilibrium in **1** when various salts are used including alternate binding of nitro group (*vide supra*). The correlations for two linear function of δ^{infl}(H1/H7)=$f(\sigma)$ and δ^{infl}(H3/H5)=$f(\sigma)$ are exemplified in Figure S16.

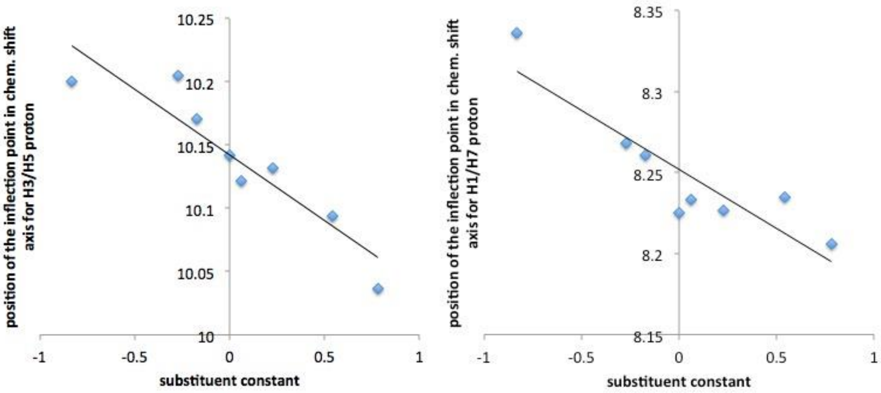

Figure S16. Correlation between substituent constant and position of the inflection point on chemical shift axis

The position of the inflection point on Y-axis for both protons (H1/H7 and H3/H5, Figure S16) is higher for benzoates carrying electron-donating substituent than that for electron acceptors. This is in agreement with the hydrogen bond accepting abilities of anions carrying electron-donating substituents. On the other hand, it is interesting that the position of inflection point on X-axis in titration curves ([**G**]:[**H**]^{infl}) is also higher for electron-donating substituents than that for acceptors (Table S1). This suggests that the structure shown in Figure 4 may be important in the beginning of the titration. Thus, most probably, at low [**G**]:[**H**] ratio the anion interacts with a dimer **1e**$_2$ forming bifurcated hydrogen-bonding complex as shown in Figure 4 and when the concentration is higher the dimer dissociate giving the opportunity to form heterocomplex. At the same time the electron donating substituent causes enhanced stabilization of **1e**···**anion** associate shown in Figure 5 (main text) by bifurcation of the hydrogen bonds. This, in turn, causes shift of the **1e**→**1a** equilibrium (or other forms) towards higher concentration of the salt shifting the ([**G**]:[**H**]^{infl}) to higher values.

After reaching the critical concentration of the salt the stability of the hydrogen-bonded complexes is driven by substituent. This is manifested by the higher CIS and δ^{infl} values for electron donating substituents than that for electron acceptors (see Figure 6 in main text and Table S1).

NMR spectra ($CDCl_3$, 20°C)

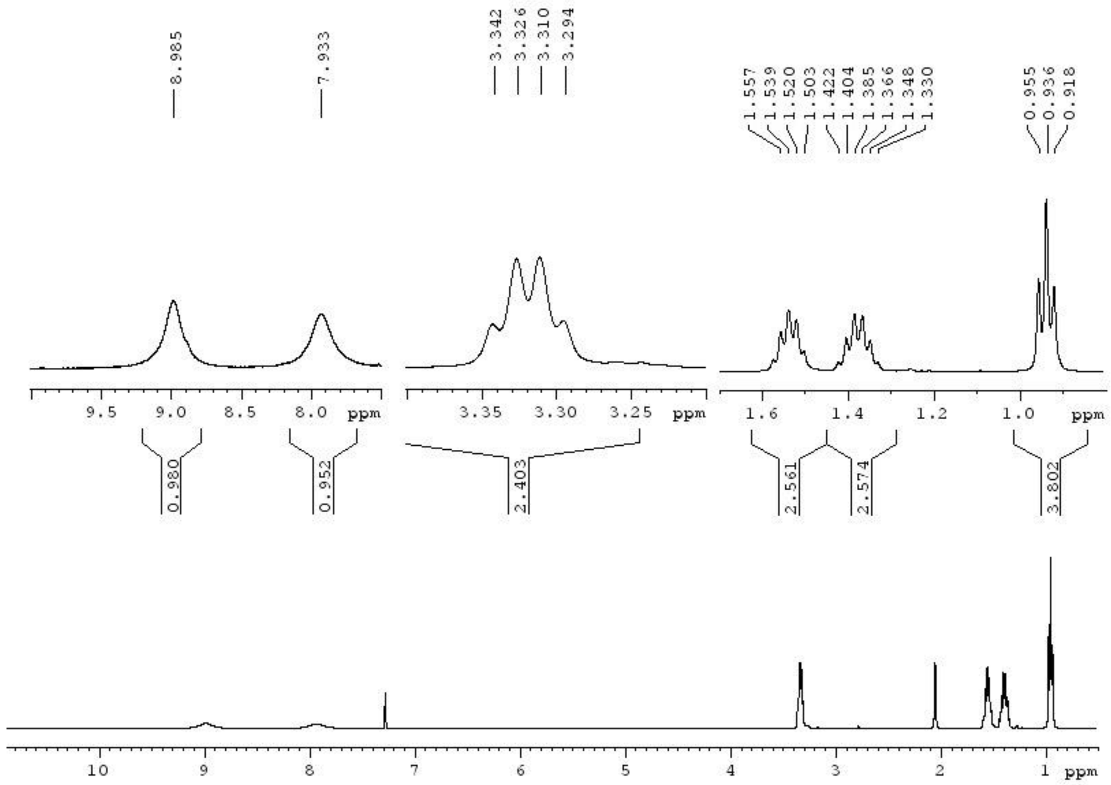

^{1}H NMR spectrum of **1**

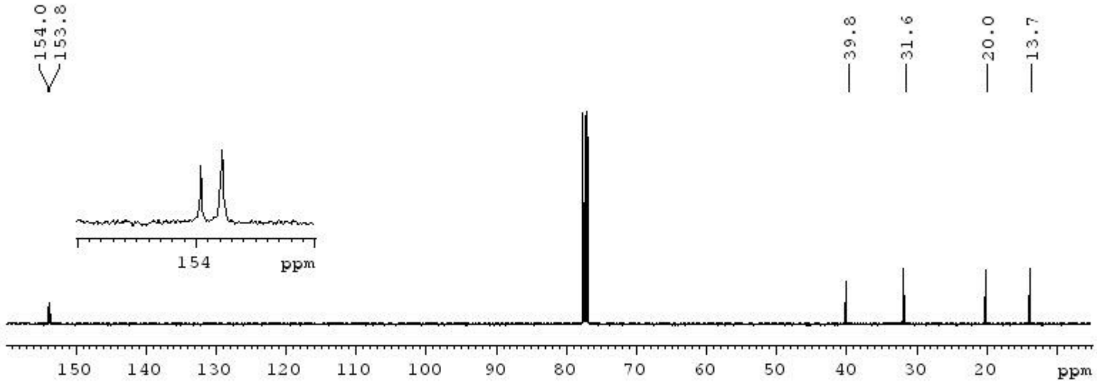

^{13}C NMR spectrum of **1**

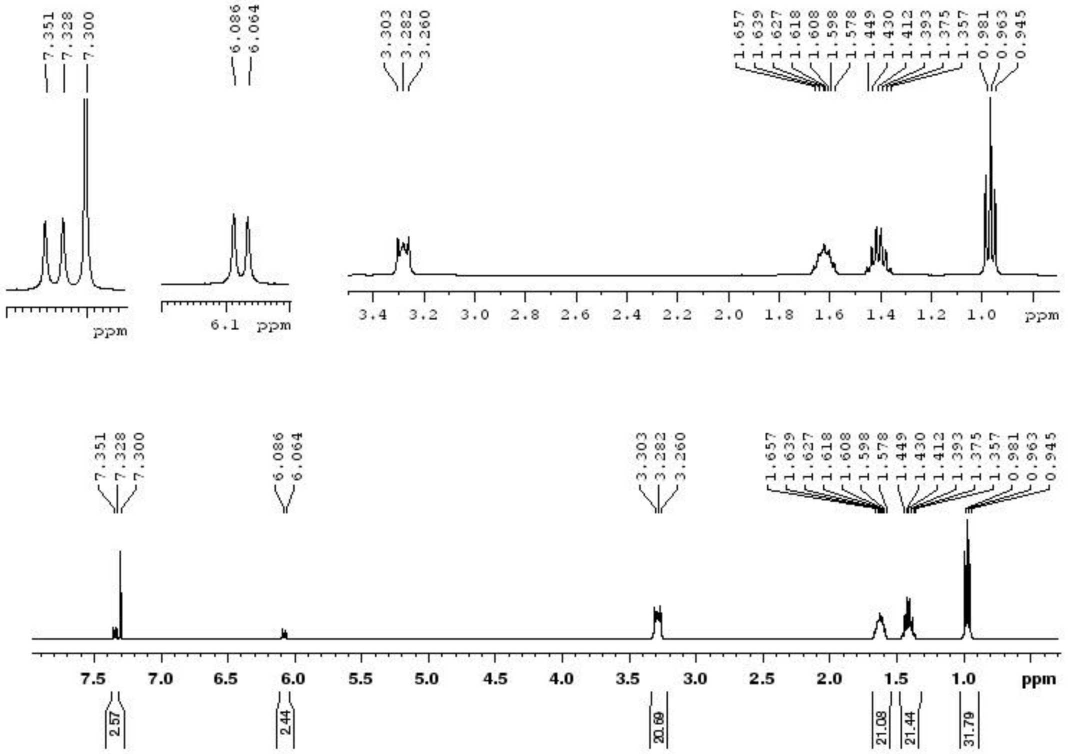

^{1}H NMR spectrum of **10**

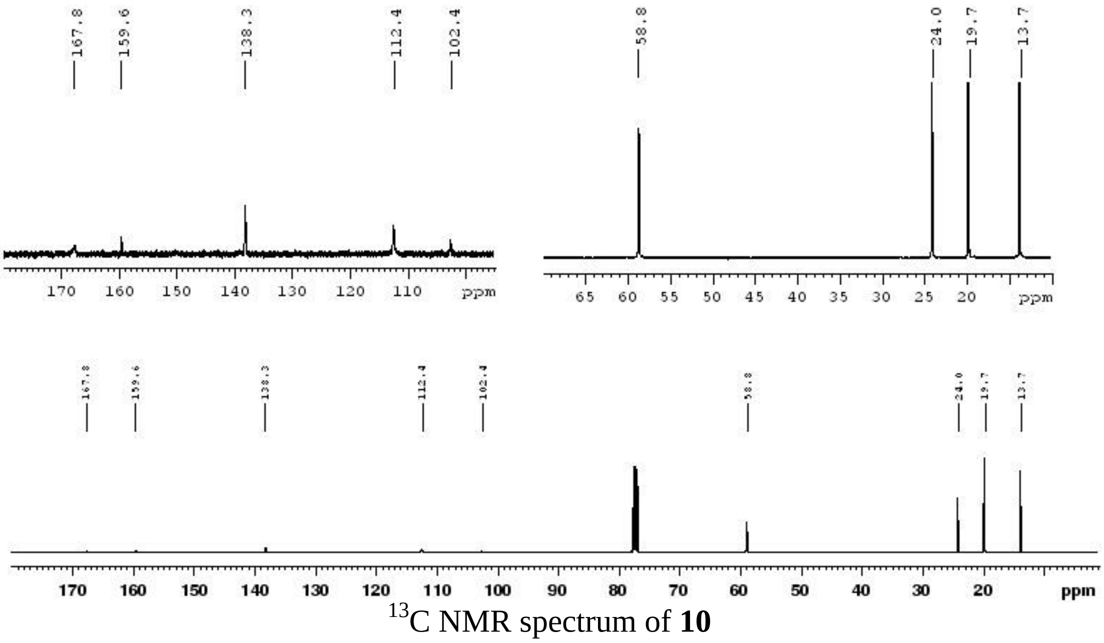

^{13}C NMR spectrum of **10**

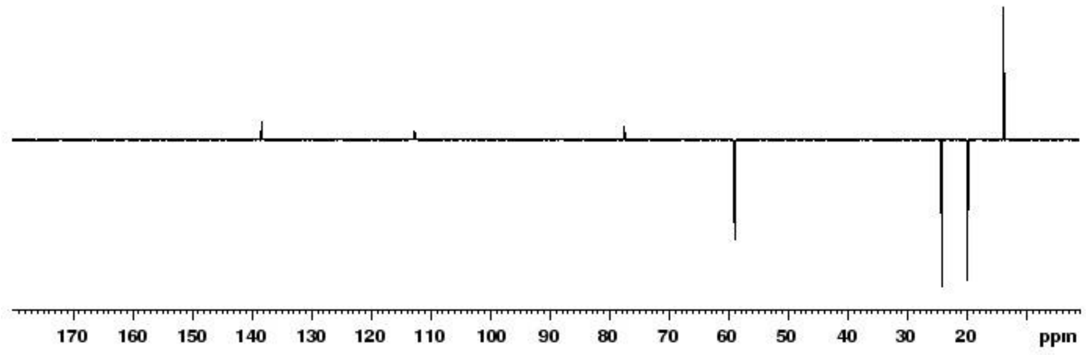

DEPT

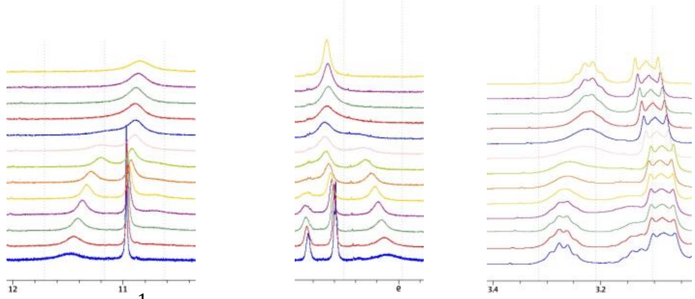

Figure S17. The results of ^{1}H VT (+20 to −40 °C) NMR experiments (**1···2** in [1]:[0.5] ratio). High temperatures at the top.

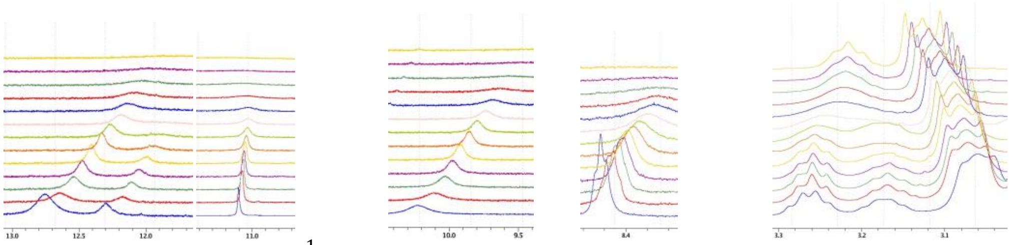

Figure S18. The results of ^{1}H VT (+20 to −40 °C) NMR experiments (**1···2** in [1]:[1] ratio)

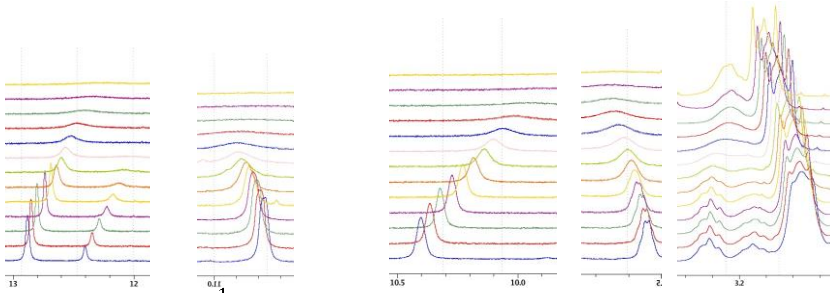

Figure S19. The results of ^{1}H VT (+20 to −40 °C) NMR experiments (**1···2** in [1]:[2] ratio)

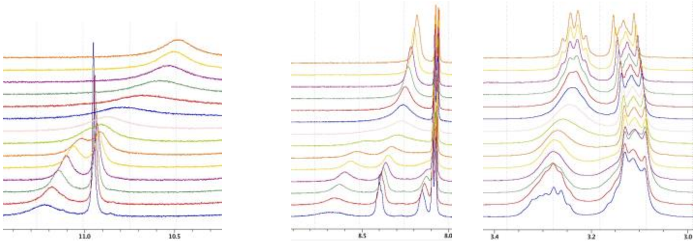

Figure S20. The results of ^{1}H VT (+20 to −40 °C) NMR experiments (**1···5** in [1]:[0.5] ratio)

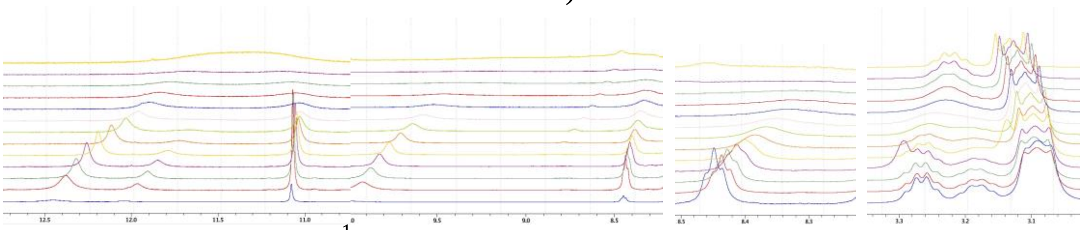

Figure S21. The results of ^{1}H VT (+20 to −40 °C) NMR experiments (**1···5** in [1]:[1] ratio)

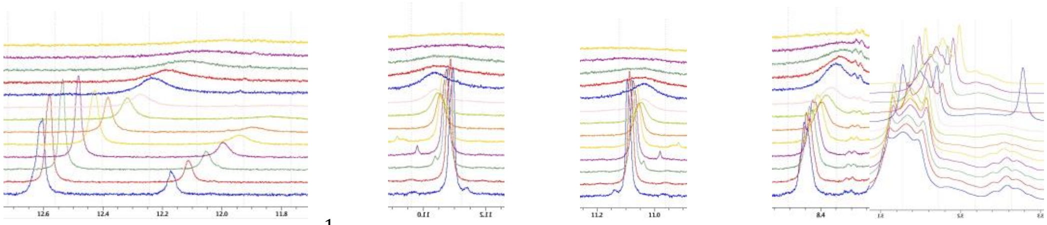

Figure S22. The results of ^{1}H VT (+20 to −40 °C) NMR experiments (**1···5** in [1]:[2] ratio)

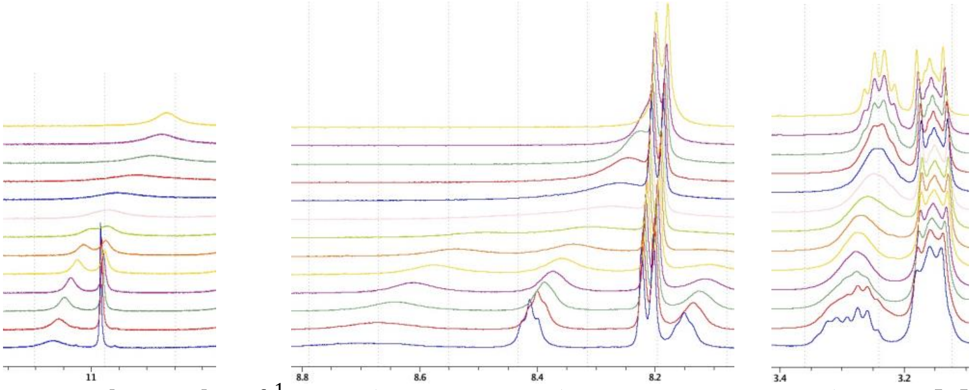

Figure S23. The results of ^{1}H VT (+20 to −40 °C) NMR experiments (**1···8** in [1]:[0.5] ratio)

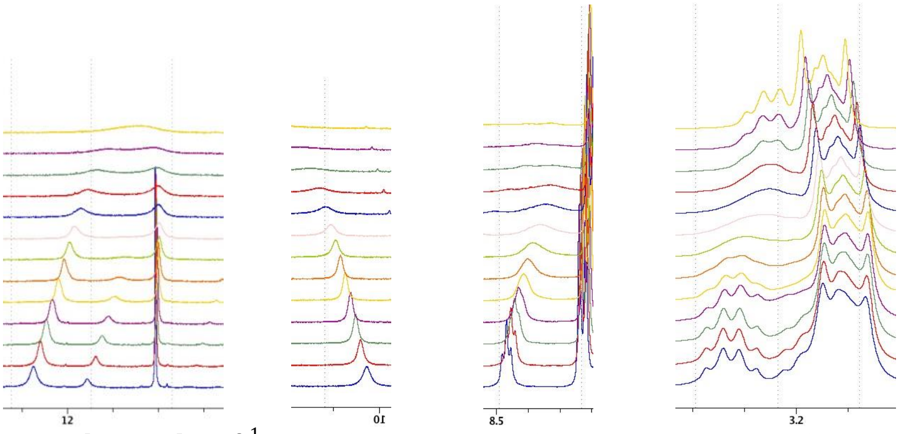

Figure S24. The results of ^{1}H VT (+20 to −40 °C) NMR experiments (**1···8** in [1]:[1] ratio)

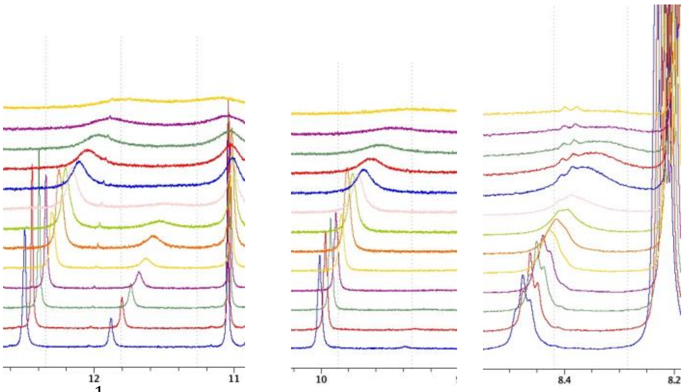

Figure S25. The results of ^{1}H VT (+20 to −40 °C) NMR experiments (**1···8** in [1]:[2] ratio)

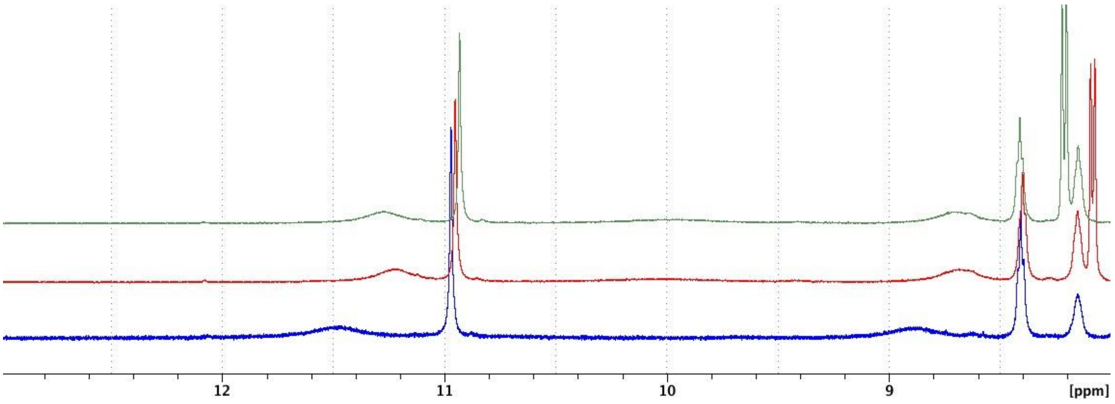

a)

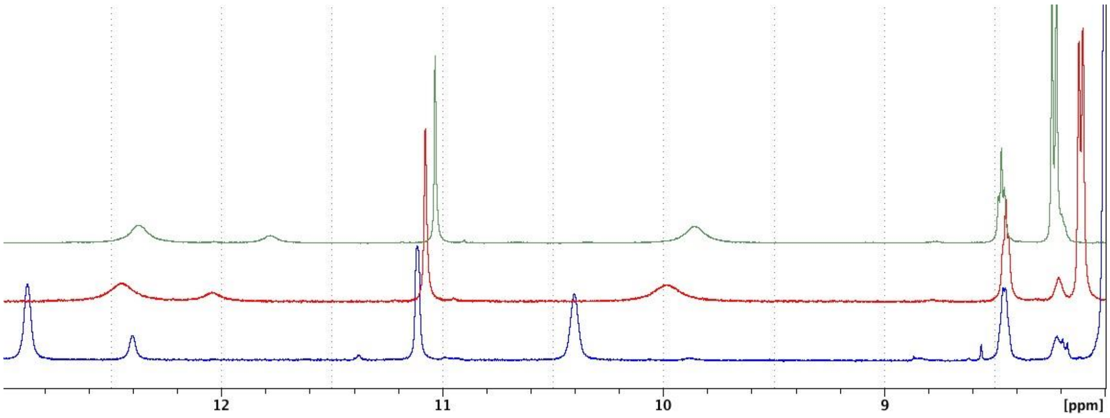

b)

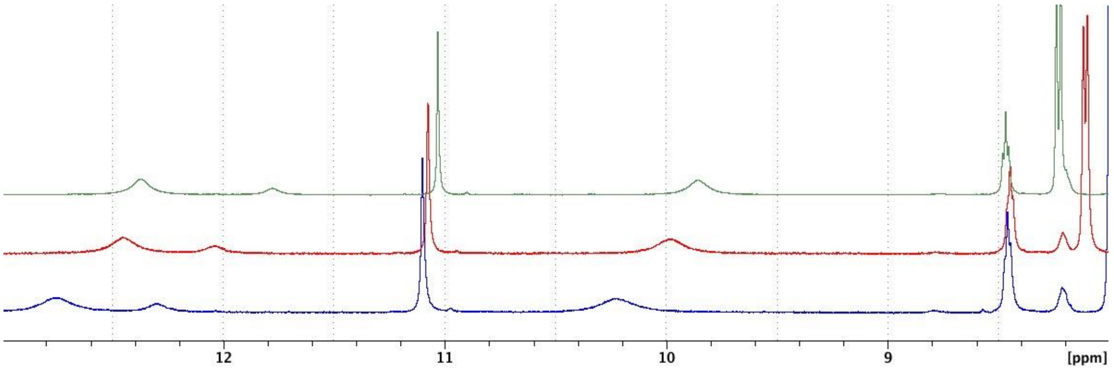

c)

Figure S26. The stacked spectra of **1···2** (bottom spectrum, blue), **1···5** (middle spectrum, red), **1···8** (top spectrum, green) in various [**G**]:[**H**] ratios *a*) 0.5, *b*) 1 and *c*) 2
at −40 °C

The NOE spectra

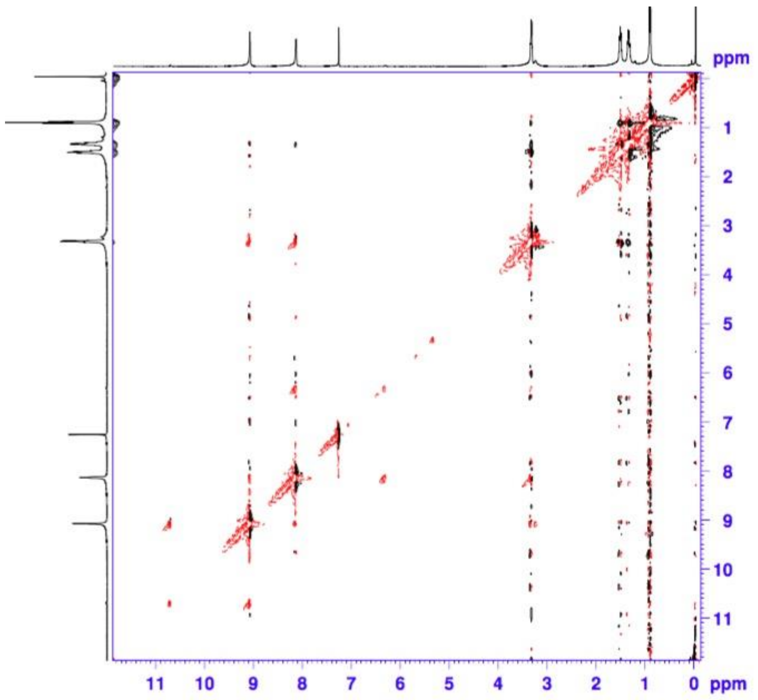

Figure S27. The NOE spectra of **1** recorded at −40 °C in $CDCl_3$

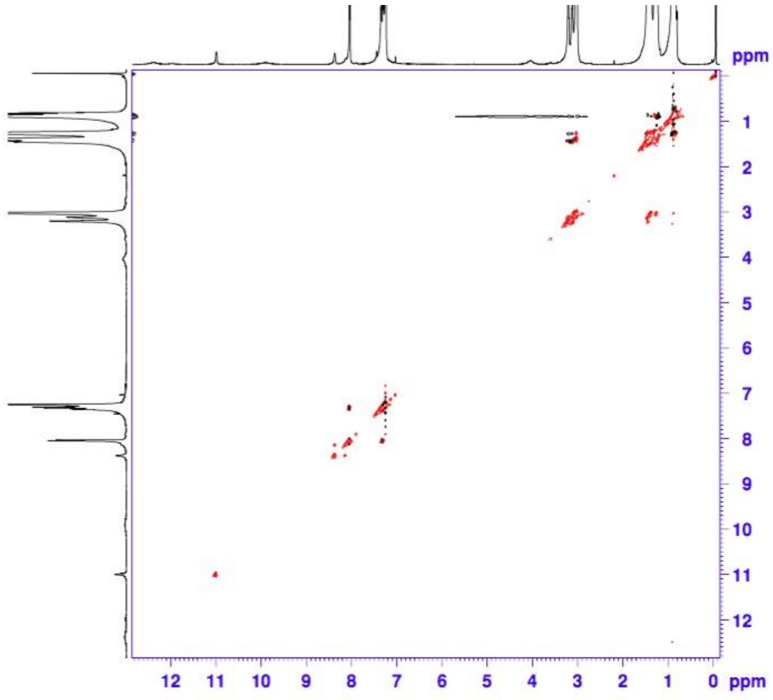

Figure S28. The NOE spectra of **1···5** recorded at −40 °C in $CDCl_3$

We would expect that NH/NH (H1/H3) NOE signals would be observed if at low temperatures **1a** form is present in its multimeric structure (Figure 4) or as associate. Since no strong cross-peak is observed we concluded 1a is not present at lowered temperatures neither in **1** nor in its complex with **5**.

For the simplicity of computations the *n*-butyl group was replaced by methyl. Such structure is labeled as **1’**.

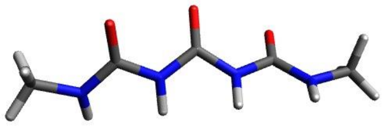

1'a

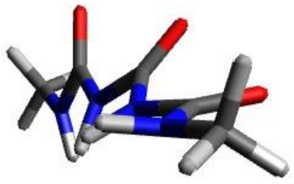

side view

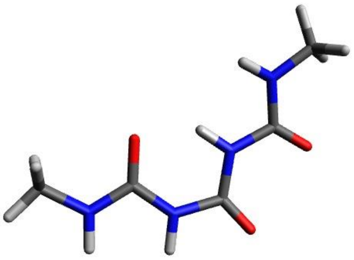

1'b

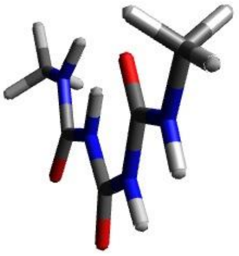

side view

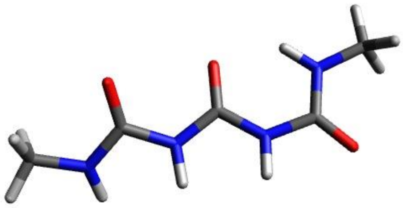

1'c

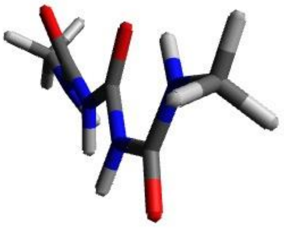

side view

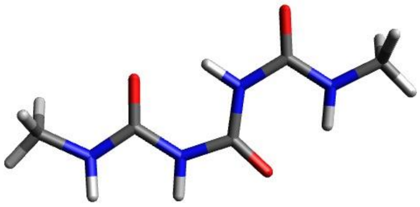

1'd

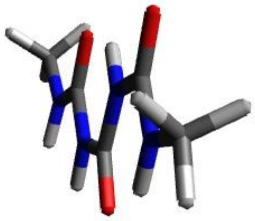

side view

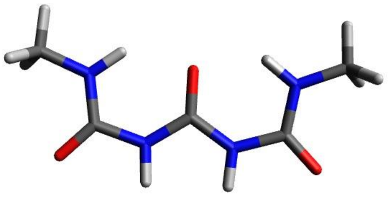

1'e

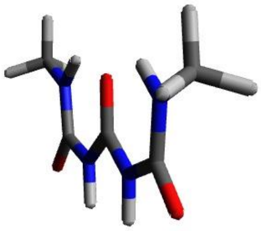

side view

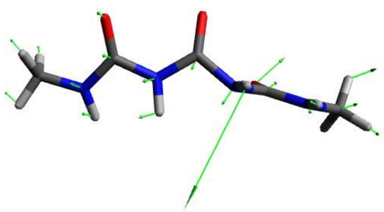

1'b-TS-1'a

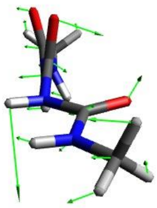

side view

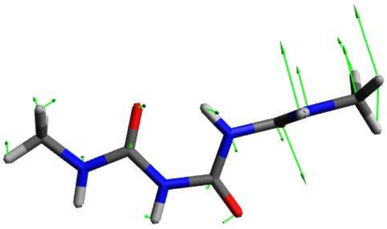

1'd-TS-1'b

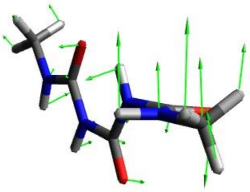

side view

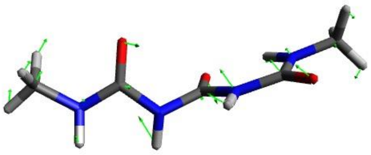

1'd-TS-1'c

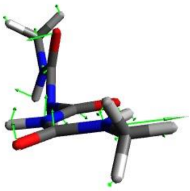

side view

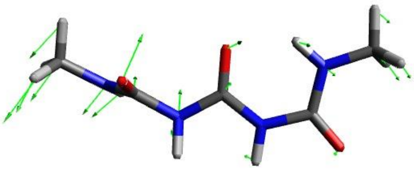

1'c-TS-1'e

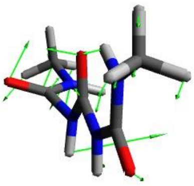

side view

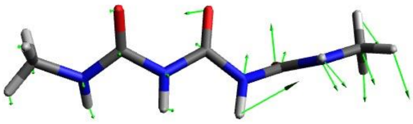

1'c-TS-1'a

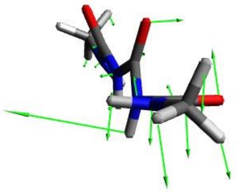

side view

Figure S29. The geometry of rotameric forms of **1'** and its transition states (imaginary frequencies shown as green arrows)

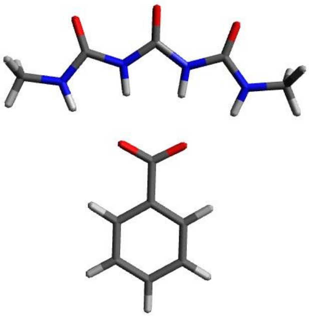

1’a···5

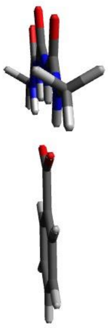

side view

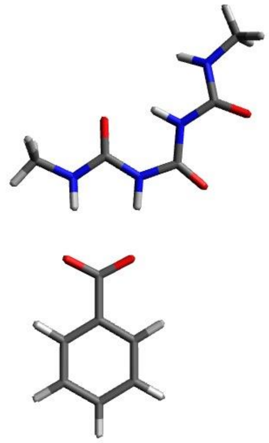

1’b···5

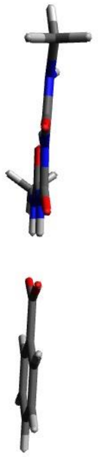

side view

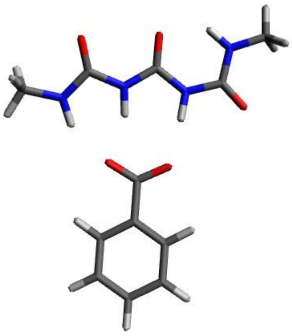

1’c···5 form 1

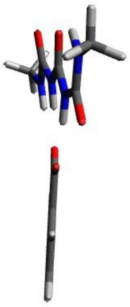

side view

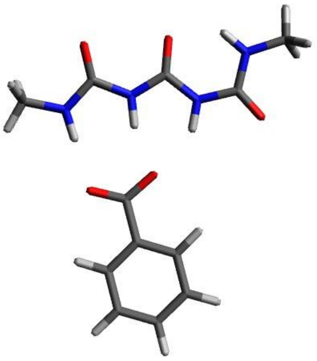

1’c···5 form 2

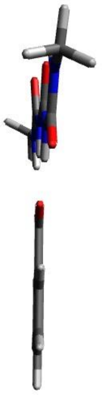

1’c···5 form 2 side view

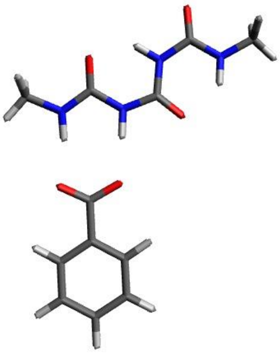

1’d···5

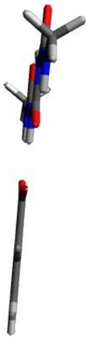

side view

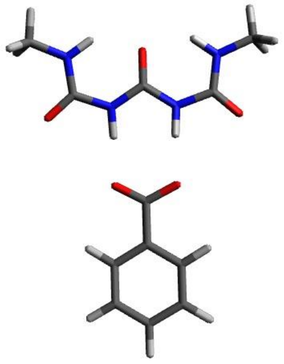

1’e···5

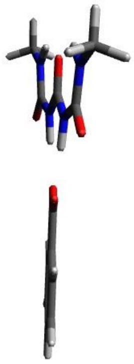

side view

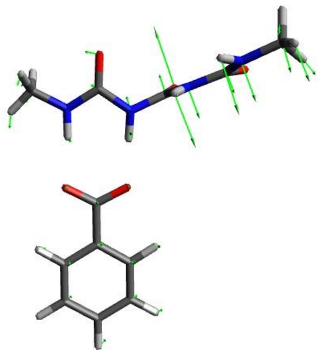

1’a···5-TS-1’b···5

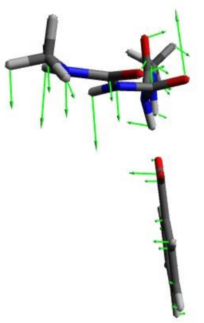

side view

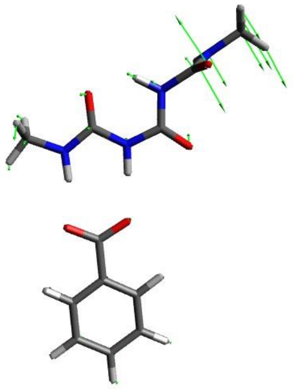

1’b···5-TS-1’d···5

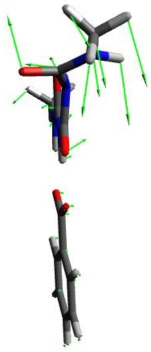

side view

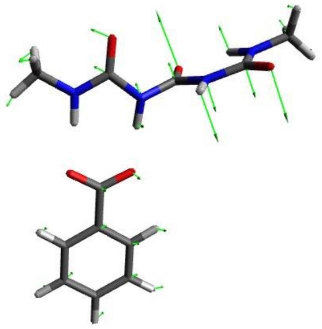

1’d···5-TS-1’c···5 (form 1)

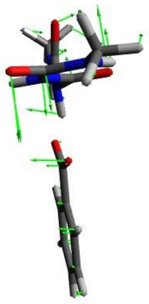

side view

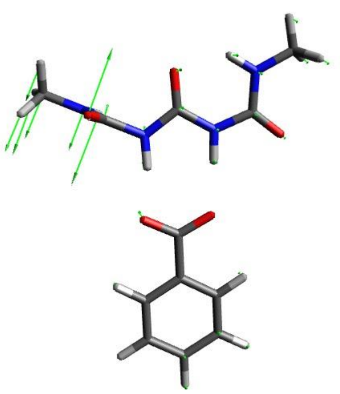

1’c···5(form 1)**-TS-1’e···5**

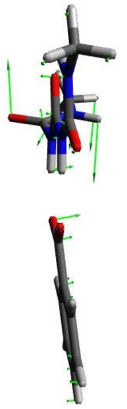

side view

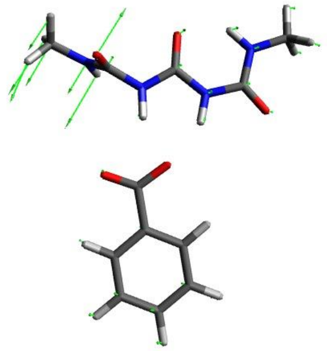

1’c···5(form 2)**-TS-1’e···5**

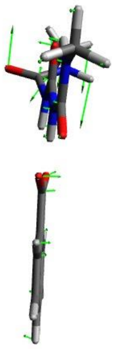

side view

Figure S30. The geometry of **1’···5** complexes

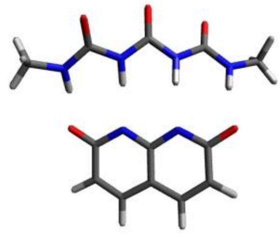

1’a···10

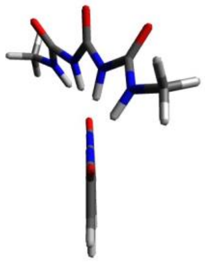

1’a···10 side view

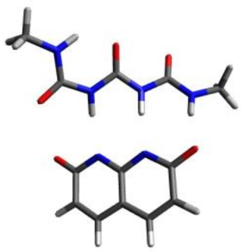

1’c···10 (form 1)

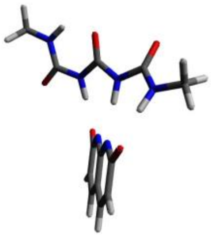

1’c···10 (form 1) side view

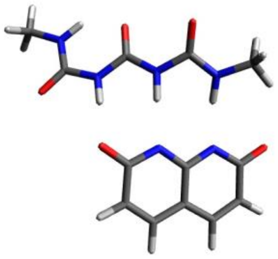

1’c···10 (form 2)

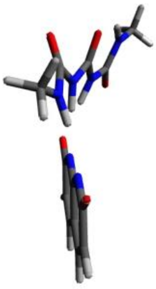

1’c···10 side view (form 2)

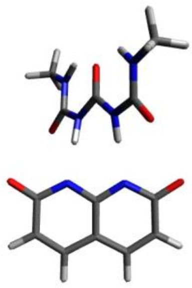

1’e···10

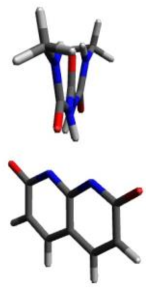

1’e···10 side view

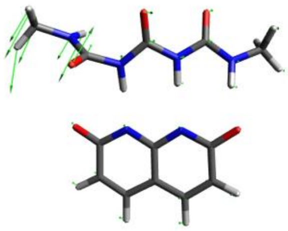

1’a···10-TS-1’c···10 (TS1)

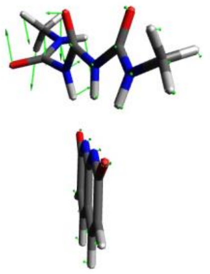

1’a···10-TS-1’c···10 (TS1) side view

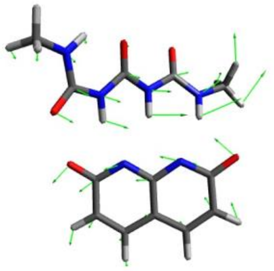

1’c···10 (form 1)-TS-1’c···10 (form 2)
(TS2)

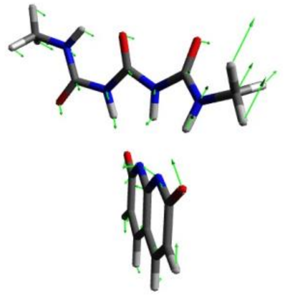

1’c···10 (form 1)-TS-1’c···10 (form 2)
(TS2) side view

Figure S31. The geometry of **1’···10** complexes

Cartesian coordinates for optimized structures

1'a E = -641.1267576 a.u.

O -2.69285 0.95314 0.92376
C -2.45346 0.01520 0.19803
N -1.14532 -0.43480 -0.07704
C 0.00000 0.35634 -0.00002
O 0.00001 1.55605 0.00000
N 1.14532 -0.43481 0.07699
C 2.45347 0.01520 -0.19804
O 2.69287 0.95315 -0.92375
N 3.40568 -0.75960 0.38492
C 4.81165 -0.45942 0.21450
N -3.40569 -0.75959 -0.38492
C -4.81166 -0.45941 -0.21445
H -1.03913 -1.37636 -0.41014
H 1.03912 -1.37638 0.41007
H 3.14634 -1.34281 1.15881
H -3.14638 -1.34279 -1.15882
H 5.10945 0.43210 0.76911
H 5.02672 -0.29687 -0.83871
H 5.39331 -1.30841 0.56449
H -5.10949 0.43210 -0.76906
H -5.02669 -0.29686 0.83877
H -5.39332 -1.30841 -0.56442

1'b E = -641.1418524 a.u.

O 1.52733 -1.24861 -0.02107
C 2.07551 -0.15285 0.00092
N 1.36847 1.04762 0.02880
C -0.01657 1.28534 0.03318
O -0.41452 2.42423 0.07346
N -0.77447 0.14476 -0.00443
C -2.17940 0.07778 -0.04323
O -2.90849 1.03896 -0.14600
N -2.62853 -1.20318 0.02290
C -4.04396 -1.49882 0.05013
N 3.41655 -0.00880 0.00247
C 4.30149 -1.15491 -0.02857
H 1.88234 1.91092 0.06211
H -0.23070 -0.71124 -0.01926
H -1.98741 -1.94439 0.23459
H 3.82207 0.90760 0.00665
H -4.55109 -0.97628 -0.75798
H -4.50369 -1.20223 0.99458
H -4.17616 -2.56877 -0.08712
H 4.14912 -1.74358 -0.93244
H 4.13867 -1.79656 0.83628
H 5.32583 -0.79421 -0.01133

1'c E = -641.1396535 a.u.

O -2.49770 -1.23234 -0.65103
C -2.39036 -0.13969 -0.14526
N -1.15515 0.53525 -0.00359
C 0.09044 -0.07067 0.00870
O 0.26515 -1.25959 0.15808
N 1.11626 0.84395 -0.14003
C 2.51310 0.63530 -0.07634
O 3.24766 1.59696 -0.22673

N 2.91917 -0.62046 0.15191
C 4.32341 -0.95620 0.22545
N -3.43532 0.60673 0.29460
C -4.79101 0.10335 0.22610
H -1.18997 1.53547 0.07800
H 0.89819 1.80258 -0.34834
H 2.20910 -1.33199 0.21885
H -3.25491 1.37766 0.91049
H 4.82221 -0.38062 1.00430
H 4.82790 -0.76315 -0.72158
H 4.40910 -2.01333 0.46056
H -4.97717 -0.67032 0.97287
H -4.97576 -0.31736 -0.75906
H -5.47767 0.92999 0.38928

1'd E = -641.151982 a.u.

O 1.83934 -1.22369 -0.04466
C 2.16203 -0.04635 -0.01871
N 1.22795 0.99563 -0.02250
C -0.16224 0.93997 -0.01710
O -0.79345 1.98369 -0.01147
N -0.70472 -0.30945 -0.01776
C -2.06726 -0.68117 -0.00187
O -2.34668 -1.86724 -0.00340
N -2.96083 0.31959 0.01490
C -4.38249 0.06018 0.03429
N 3.44558 0.36953 0.00326
C 4.54378 -0.57396 0.05035
H 1.55822 1.94518 -0.03232
H -0.05022 -1.08276 -0.03076
H -2.60367 1.26114 0.01107
H 3.65305 1.34389 0.11499
H -4.66655 -0.51245 0.91708
H -4.69461 -0.49578 -0.84975
H -4.90336 1.01358 0.05160
H 4.42312 -1.32872 -0.72324
H 4.60321 -1.07304 1.01812
H 5.46841 -0.03219 -0.12765

1'e E = -641.1516356 a.u.

O 3.36707 -1.38838 -0.01000
C 2.50257 -0.53046 -0.00432
N 1.14882 -0.94880 -0.00580
C 0.00000 -0.19318 0.00000
O 0.00000 1.02877 0.00000
N -1.14882 -0.94880 0.00580
C -2.50257 -0.53046 0.00433
O -3.36707 -1.38838 0.01000
N -2.72162 0.79062 -0.00417
C -4.06360 1.32989 -0.00781
N 2.72162 0.79062 0.00417
C 4.06360 1.32989 0.00781
H 1.07758 -1.95146 -0.01237
H -1.07758 -1.95146 0.01237
H -1.91862 1.39752 -0.00511
H 1.91862 1.39752 0.00511
H -4.61360 1.02248 0.88127

H -4.61695 0.99974 -0.88648
H -3.99504 2.41393 -0.02198
H 4.61360 1.02248 -0.88127
H 4.61695 0.99974 0.88648
H 3.99504 2.41393 0.02197

1'b-TS-1'a E = -641.1203002 a.u., imaginary frequency -91.97 cm^{-1}

O -2.10365 -0.58729 1.18591
C -2.28544 -0.20069 0.04533
N -1.21683 0.15245 -0.78292
C -0.02917 0.67946 -0.14921
O -0.01934 1.68527 0.49972
N 1.04495 -0.12718 -0.41841
C 2.39801 0.14679 -0.09471
O 2.80794 1.25862 0.14283
N 3.17083 -0.96598 -0.13893
C 4.58355 -0.90582 0.17150
N -3.50499 -0.15131 -0.54594
C -4.70643 -0.51343 0.17405
H -1.43796 0.63427 -1.64017
H 0.82715 -0.99022 -0.88436
H 2.72801 -1.86629 -0.12759
H -3.60462 0.29331 -1.43847
H 4.76042 -0.80579 1.24358
H 5.03138 -0.05374 -0.33320
H 5.05783 -1.81599 -0.18600
H -4.97055 0.23231 0.92536
H -4.56910 -1.47046 0.67245
H -5.52297 -0.60394 -0.53764

1'd-TS-1'b E = -641.13719 a.u., imaginary frequency -53.53 cm^{-1}

O -1.73444 -1.26266 0.07255
C -2.14692 -0.11771 -0.05598
N -1.30015 0.98358 -0.16036
C 0.09644 1.03173 -0.09438
O 0.66007 2.10789 -0.13946
N 0.72538 -0.16812 -0.02222
C 2.11236 -0.23711 0.36631
O 2.47878 -0.04646 1.50392
N 2.91102 -0.57475 -0.65719
C 4.34396 -0.71110 -0.51077
N -3.46199 0.18371 -0.12289
C -4.47762 -0.83839 0.02169
H -1.69951 1.90036 -0.25998
H 0.13233 -0.97889 0.12165
H 2.49776 -0.64059 -1.56838
H -3.75369 1.14249 -0.14455
H 4.57155 -1.29760 0.37660
H 4.82754 0.26200 -0.41709
H 4.73779 -1.22181 -1.38506
H -4.48653 -1.25491 1.02924
H -4.30641 -1.64661 -0.68625
H -5.44529 -0.39002 -0.18488

1'd-TS-1'c E = -641.1317547 a.u., imaginary frequency =118.50 cm^{-1}

O 2.04024 -0.50177 1.19580
C 2.25826 -0.01426 0.10288
N 1.21633 0.42333 -0.72233

C -0.07814 -0.15834 -0.51152
O -0.30591 -1.33629 -0.65007
N -0.99758 0.78569 -0.17142
C -2.38782 0.64448 0.08821
O -3.02740 1.64289 0.36176
N -2.87621 -0.59870 0.00391
C -4.26943 -0.88093 0.27053
N 3.50109 0.18644 -0.40420
C 4.68109 -0.02005 0.40893
H 1.42566 0.56925 -1.69756
H -0.66749 1.72864 -0.05937
H -2.22992 -1.34181 -0.20672
H 3.60281 0.73432 -1.23801
H -4.91630 -0.30881 -0.39312
H -4.53076 -0.63885 1.30069
H -4.44056 -1.94039 0.10311
H 4.62282 -0.98622 0.90331
H 4.79511 0.75383 1.17070
H 5.55403 -0.01133 -0.23880

1'c-TS-1'e E = -641.1362105 a.u., imaginary frequency -76.87cm^{-1}

O -2.56670 0.22393 1.46123
C -2.37479 -0.17463 0.33659
N -1.14297 -0.83311 -0.05595
C 0.03642 -0.13062 -0.01803
O 0.06888 1.08660 -0.03398
N 1.16302 -0.92869 -0.01012
C 2.52387 -0.55163 -0.04559
O 3.36841 -1.43160 -0.05011
N 2.77741 0.76374 -0.07155
C 4.13058 1.27133 -0.09193
N -3.25318 -0.11403 -0.67378
C -4.56339 0.48372 -0.52469
H -1.12525 -1.82492 0.11305
H 1.05767 -1.92777 0.00599
H 1.98601 1.38702 -0.04923
H -2.94041 -0.40319 -1.58173
H 4.67986 0.88668 -0.95043
H 4.67181 0.99629 0.81368
H 4.08728 2.35474 -0.15914
H -4.49344 1.56669 -0.42175
H -5.05779 0.08251 0.35742
H -5.15649 0.24613 -1.40320

1'c-TS-1'a E = -641.1229297 a.u., imaginary frequency -83.70 cm^{-1}

O 2.56361 1.40254 -0.03429
C 2.42103 0.20408 -0.12029
N 1.16935 -0.42247 -0.28694
C -0.05782 0.12039 0.08292
O -0.19447 1.04644 0.84488
N -1.12658 -0.50163 -0.52855
C -2.43169 -0.43862 0.09352
O -2.66997 -0.96731 1.15521
N -3.32009 0.22403 -0.66114
C -4.69274 0.42082 -0.24575
N 3.44986 -0.68544 -0.13310
C 4.81766 -0.23895 0.02634
H 1.17179 -1.34063 -0.69299
H -0.97171 -1.30329 -1.11609
H -2.97309 0.70993 -1.46708

H 3.25267 -1.64997 0.05918
H -4.75757 1.12523 0.58393
H -5.12564 -0.52620 0.06921
H -5.25989 0.80756 -1.08748
H 5.01509 0.12147 1.03723
H 5.02747 0.56659 -0.67316
H 5.48113 -1.07179 -0.19148

1’a···5 E = -1061.3589656 a.u.

O -3.49012 -2.87790 0.02212
C -2.34328 -2.47145 0.00735
N -1.95388 -1.13104 -0.00004
C -2.76280 -0.00106 -0.04182
O -3.96686 -0.00181 -0.10910
N -1.95523 1.12992 -0.00027
C -2.34612 2.46992 0.00358
O -3.49346 2.87517 0.01230
N -1.26325 3.29658 0.00208
C -1.40808 4.73064 0.02814
N -1.25959 -3.29699 0.00181
C -1.40295 -4.73110 0.03246
H -0.94230 -0.98573 0.01849
H -0.94357 0.98567 0.02140
H -0.34202 2.89084 0.00639
H -0.41651 5.17675 0.02037
H -1.95890 5.09178 -0.84149
H -1.93351 5.06481 0.92421
H -0.33878 -2.89027 0.00304
H -0.41103 -5.17633 0.02128
H -1.92400 -5.06336 0.93181
H -1.95741 -5.09505 -0.83366
O 0.89138 -1.11452 0.02557
C 1.46762 0.00065 0.02329
O 0.89061 1.11538 0.03336
C 2.98511 0.00108 0.00636
C 3.69423 1.20069 0.00649
C 5.08287 1.20393 -0.01072
C 5.78104 0.00161 -0.02877
C 5.08311 -1.20100 -0.02896
C 3.69449 -1.19826 -0.01132
H 3.13979 2.12917 0.01996
H 5.62174 2.14345 -0.01041
H 6.86400 0.00181 -0.04267
H 5.62215 -2.14032 -0.04287
H 3.14018 -2.12690 -0.01129

1’b···5 E = -1061.3589554 a.u.

O -4.53507 -2.32381 -0.19986
C -4.51819 -1.11727 -0.06917
N -3.37128 -0.32660 0.00440
C -2.05533 -0.74869 0.05014
O -1.68735 -1.90086 0.09231
N -1.12987 0.28922 0.05287
C -1.35276 1.66607 0.02586
O -2.48241 2.16854 0.01581
N -0.23299 2.40174 0.01567
C -0.29139 3.84273 -0.01009
N -5.65682 -0.36507 -0.00785
C -6.96194 -0.98348 0.00504

H -3.46367 0.68543 -0.00072
H -0.15207 -0.03328 0.08134
H 0.69673 1.96915 0.00287
H -5.59119 0.59911 0.25921
H -0.81028 4.23958 0.86389
H -0.80059 4.21031 -0.90258
H 0.72918 4.21804 -0.01074
H -7.15169 -1.52639 0.93362
H -7.05454 -1.68247 -0.82366
H -7.71453 -0.20700 -0.10922
C 4.14392 -1.68600 0.06954
C 3.94788 -0.30792 0.00378
C 5.06222 0.52450 -0.08329
C 6.34591 -0.00586 -0.10365
C 6.53045 -1.38265 -0.03730
C 5.42526 -2.22225 0.04909
C 2.54391 0.27903 0.02606
O 1.58878 -0.52727 0.11259
O 2.44787 1.52845 -0.04363
H 3.27645 -2.32861 0.13619
H 4.90236 1.59315 -0.13541
H 7.20316 0.65302 -0.17150
H 7.53022 -1.79927 -0.05328
H 5.56296 -3.29549 0.10054

1'c (form 1)+5 E = -1061.3649361 a.u.

O 2.09195 -3.19793 -0.06934
C 2.70003 -2.13681 -0.04749
N 1.98403 -0.93319 -0.01663
C 2.48656 0.35667 0.00185
O 3.67592 0.62171 0.02046
N 1.47408 1.28981 -0.01221
C 1.60462 2.68512 0.07387
O 2.65094 3.28261 0.24160
N 0.40034 3.29798 -0.05087
C 0.26942 4.72720 0.08038
N 4.04396 -2.04432 -0.05219
C 4.88038 -3.21846 -0.07373
H 0.96640 -1.06106 0.01356
H 0.51133 0.95935 -0.10646
H -0.42511 2.72178 -0.12965
H 4.43706 -1.11688 -0.01830
H 0.92687 5.24616 -0.61700
H 0.51031 5.06801 1.08974
H -0.75899 5.00073 -0.14276
H 4.68991 -3.82638 -0.95889
H 4.71789 -3.84162 0.80693
H 5.91952 -2.89900 -0.08758
C -3.58025 -1.83415 0.26126
C -3.15797 -0.53559 -0.01642
C -4.11768 0.44501 -0.25993
C -5.47157 0.13626 -0.22905
C -5.88244 -1.16259 0.04993
C -4.93268 -2.14803 0.29609
C -1.67701 -0.19929 -0.05335
O -0.86813 -1.13320 0.13892
O -1.36729 0.99969 -0.27747
H -2.83020 -2.59058 0.44737

H -3.78529 1.45162 -0.47480
H -6.20719 0.90765 -0.42199
H -6.93769 -1.40559 0.07520
H -5.24711 -3.16139 0.51388

1'c (form 2)+5 E = -1061.3664127 a.u.

O 2.06476 -3.24974 0.03208
C 2.70130 -2.20559 -0.00065
N 2.01510 -0.98532 0.00324
C 2.53293 0.30709 -0.01772
O 3.72589 0.54753 -0.05468
N 1.52171 1.23298 0.00408
C 1.64022 2.63400 0.03643
O 2.69741 3.23524 0.10525
N 0.42910 3.23199 -0.01261
C 0.30806 4.66709 0.03634
N 4.04452 -2.14136 -0.04275
C 4.85827 -3.33229 -0.02830
H 1.00818 -1.08958 0.02929
H 0.57398 0.84088 0.00752
H -0.42158 2.66925 -0.05548
H 4.45608 -1.22109 -0.05275
H 0.84746 5.14252 -0.78436
H 0.69308 5.07426 0.97337
H -0.74593 4.92261 -0.04597
H 4.58883 -4.00295 -0.84432
H 4.75379 -3.87955 0.91013
H 5.89816 -3.03844 -0.14644
C -3.22311 -1.75091 0.06572
C -3.22177 -0.35971 -0.01374
C -4.44149 0.31153 -0.07203
C -5.63943 -0.39068 -0.05035
C -5.63016 -1.77903 0.02998
C -4.41824 -2.45832 0.08785
C -1.91495 0.41376 -0.03335
O -0.85352 -0.25938 0.03258
O -1.98200 1.66048 -0.11185
H -2.27338 -2.26652 0.10911
H -4.43262 1.39128 -0.13370
H -6.58084 0.14326 -0.09560
H -6.56314 -2.32902 0.04736
H -4.40516 -3.53957 0.15011

1’d···5 E = -1061.3710678 a.u.

O -2.42829 2.52822 -0.02262
C -1.33296 1.96484 -0.01994
N -1.19110 0.57203 -0.01628
C -2.17669 -0.38860 -0.00787
O -1.87823 -1.57521 -0.00902
N -3.47156 0.07274 0.00229
C -4.66552 -0.66134 0.01666
O -5.73257 -0.06381 0.02536
N -4.54857 -2.00078 0.02021
C -5.70671 -2.86062 0.03472
N -0.16570 2.62385 -0.02181
C -0.12871 4.06616 -0.02598

H -0.23542 0.18254 -0.02185
H -3.57524 1.08186 -0.00109
H -3.61246 -2.37560 0.01112
H 0.73182 2.12819 -0.00787
H -6.33166 -2.70077 -0.84463
H -6.31741 -2.68982 0.92195
H -5.36678 -3.89307 0.03845
H -0.60396 4.48405 0.86303
H -0.63037 4.47739 -0.90313
H 0.91404 4.37448 -0.04266
C 3.89216 -1.79691 -0.08044
C 3.80420 -0.40879 0.00245
C 4.98015 0.33352 0.08980
C 6.21847 -0.29604 0.09538
C 6.29496 -1.68201 0.01020
C 5.12735 -2.43210 -0.07860
C 2.44718 0.27937 -0.00332
O 1.43525 -0.46034 -0.03221
O 2.44091 1.53356 0.02058
H 2.97448 -2.36520 -0.14598
H 4.90651 1.41065 0.15380
H 7.12484 0.29320 0.16537
H 7.25960 -2.17483 0.01306
H 5.18078 -3.51199 -0.14547

1'e···5 E = -1061.3656134 a.u.

O 1.16847 -3.39699 -0.01642
C 1.97281 -2.47841 -0.03156
N 1.50627 -1.15123 0.00014
C 2.25428 0.00000 0.00000
O 3.48694 0.00000 0.00000
N 1.50627 1.15123 -0.00014
C 1.97281 2.47841 0.03156
O 1.16846 3.39699 0.01641
N 3.30713 2.66282 0.08238
C 3.87882 3.98653 0.11157
N 3.30713 -2.66282 -0.08238
C 3.87883 -3.98653 -0.11157
H 0.47501 -1.09245 0.06166
H 0.47501 1.09245 -0.06166
H 3.88688 1.84025 0.06660
H 3.88688 -1.84025 -0.06659
H 3.53702 4.54714 0.98224
H 3.61580 4.55579 -0.78107
H 4.96094 3.89224 0.16004
H 3.53702 -4.54713 -0.98223
H 3.61580 -4.55579 0.78107
H 4.96094 -3.89224 -0.16004
C -4.09591 -1.17936 0.21820
C -3.38662 0.00000 -0.00000
C -4.09591 1.17936 -0.21820
C -5.48505 1.18265 -0.21804
C -6.18360 -0.00000 0.00000
C -5.48505 -1.18265 0.21805
C -1.86548 0.00000 -0.00000
O -1.29360 -1.09192 0.22750
O -1.29361 1.09192 -0.22751
H -3.53834 -2.09077 0.38649
H -3.53835 2.09077 -0.38649

H -6.02403 2.10686 -0.38818
H -7.26688 -0.00000 0.00001
H -6.02403 -2.10686 0.38818

1’a···5-TS-**1’b···5** E = -1061.3375098 a.u., imaginary frequency -45.17cm^{-1}
O -4.42607 -1.79764 1.11116
C -4.10830 -1.38694 0.01745
N -3.01706 -0.53001 -0.21263
C -2.27366 0.15727 0.72710
O -2.47674 0.15226 1.91033
N -1.20734 0.85848 0.09493
C -1.28717 2.24286 0.02441
O -2.36359 2.83405 0.08034
N -0.10020 2.85987 -0.15480
C -0.02245 4.28385 -0.35621
N -4.74454 -1.73577 -1.13484
C -5.92915 -2.56408 -1.10902
H -2.72546 -0.37058 -1.16026
H -0.27583 0.42687 0.21237
H 0.76224 2.31483 -0.20232
H -4.56049 -1.21426 -1.97160
H -0.45754 4.83222 0.48054
H -0.53767 4.59660 -1.26728
H 1.02708 4.55741 -0.44069
H -6.81389 -2.00631 -0.79455
H -5.78043 -3.38934 -0.41766
H -6.09783 -2.96622 -2.10516
C 3.68264 -1.88030 0.33206
C 3.64913 -0.53900 -0.04406
C 4.83647 0.07814 -0.43249
C 6.03205 -0.62879 -0.44966
C 6.05435 -1.96741 -0.07344
C 4.87555 -2.59204 0.31980
C 2.33970 0.23847 -0.03478
O 1.30377 -0.40310 0.26567
O 2.39608 1.45475 -0.32974
H 2.75769 -2.35263 0.63459
H 4.80296 1.11994 -0.72097
H 6.94748 -0.13700 -0.75577
H 6.98571 -2.52046 -0.08538
H 4.88726 -3.63380 0.61635

1’b···5-TS-**1’d···5** E = -1060.3535278 a.u., imaginary frequency -54.50cm^{-1}
O 2.40684 2.43629 -0.06486
C 1.30829 1.87337 -0.04530
N 1.16342 0.48696 -0.00359
C 2.16221 -0.47048 0.05640
O 1.88986 -1.65337 0.16780
N 3.44473 -0.00846 -0.06654
C 4.54899 -0.82974 0.32789
O 4.78317 -1.12144 1.48147
N 5.31494 -1.19288 -0.71719
C 6.48861 -2.02305 -0.56837
N 0.14621 2.54338 -0.06451
C 0.12082 3.98496 -0.09442
H 0.20940 0.10104 -0.00187
H 3.54573 1.00274 -0.03190
H 4.97853 -0.96878 -1.63472
H -0.75514 2.05697 -0.02240

H 6.22187 -3.04452 -0.29328
H 7.13970 -1.61809 0.20411
H 7.02777 -2.03911 -1.51175
H 0.65092 4.37650 -0.96358
H 0.57252 4.41756 0.80037
H -0.91858 4.30035 -0.14716
C -3.99102 -1.80232 -0.12138
C -3.87243 -0.41815 -0.01520
C -5.03159 0.34839 0.08896
C -6.28396 -0.25298 0.08624
C -6.39130 -1.63512 -0.02390
C -5.24063 -2.40949 -0.12738
C -2.50098 0.24356 -0.01684
O -1.50378 -0.51284 -0.07305
O -2.47346 1.49694 0.03618
H -3.08652 -2.39005 -0.19762
H -4.93281 1.42203 0.17258
H -7.17666 0.35497 0.16914
H -7.36654 -2.10642 -0.02776
H -5.31848 -3.48653 -0.21198

1’d···5-TS-**1'c (form 1)+5** E = -1061.3502835 a.u., imaginary frequency -50.40cm^{-1}
O 2.39125 2.79749 -0.09509
C 1.30524 2.23162 -0.00065
N 1.20855 0.84897 0.12291
C 2.29653 0.06903 -0.33047
O 2.57197 -0.10005 -1.49951
N 2.98397 -0.50631 0.70580
C 4.11040 -1.35798 0.68205
O 4.57921 -1.74554 1.73954
N 4.57713 -1.67759 -0.53358
C 5.73712 -2.52102 -0.70065
N 0.11983 2.87340 0.04102
C 0.04686 4.31227 0.02295
H 0.28040 0.40786 -0.00469
H 2.66840 -0.28283 1.63340
H 4.10729 -1.26956 -1.32703
H -0.75127 2.34225 0.09156
H 5.59398 -3.48426 -0.21174
H 6.63135 -2.05531 -0.28479
H 5.88963 -2.68619 -1.76369
H 0.50064 4.72954 -0.87762
H 0.54531 4.75595 0.88749
H -1.00273 4.59766 0.04588
C -3.64092 -1.91127 -0.10494
C -3.63700 -0.52223 0.00603
C -4.85583 0.14541 0.10701
C -6.05363 -0.55807 0.09653
C -6.04591 -1.94450 -0.01309
C -4.83495 -2.62068 -0.11358
C -2.32440 0.24941 0.01648
O -1.27272 -0.42364 -0.11528
O -2.39187 1.49223 0.15624
H -2.69089 -2.42208 -0.18316
H -4.84642 1.22350 0.19260
H -6.99437 -0.02652 0.17436
H -6.97897 -2.49474 -0.02025
H -4.82258 -3.70046 -0.19850

1'c (form 1)+5-TS-**1’e···5** E = -1061.3535293 a.u., imaginary frequency -45.58cm^{-1}

O 1.47785 -3.40999 -0.16695
C 2.20440 -2.42980 -0.07194
N 1.63615 -1.15729 0.04426
C 2.28387 0.06112 0.10399
O 3.50711 0.18956 0.08182
N 1.44225 1.12583 0.16761
C 1.96718 2.44172 0.36424
O 2.15803 2.91837 1.46409
N 2.17991 3.09891 -0.78985
C 2.70331 4.44482 -0.83396
N 3.55133 -2.49674 -0.06853
C 4.24562 -3.75588 -0.16590
H 0.60870 -1.16317 0.08508
H 0.41481 1.02740 0.25136
H 1.99514 2.60947 -1.64460
H 4.04816 -1.62256 0.00764
H 3.71688 4.48537 -0.43376
H 2.07639 5.11981 -0.25235
H 2.71780 4.77936 -1.86766
H 4.02173 -4.26707 -1.10340
H 3.97804 -4.42295 0.65460
H 5.31470 -3.56271 -0.12199
C -3.97027 -1.37017 -0.02510
C -3.34820 -0.12548 0.05330
C -4.13653 1.02257 0.01186
C -5.51766 0.93197 -0.10730
C -6.12849 -0.31494 -0.18630
C -5.35077 -1.46698 -0.14373
C -1.83477 -0.02537 0.17251
O -1.19165 -1.09933 0.18668
O -1.34149 1.12762 0.24198
H -3.35279 -2.25757 0.00756
H -3.64706 1.98503 0.07479
H -6.11867 1.83273 -0.13913
H -7.20505 -0.38849 -0.27972
H -5.82132 -2.44085 -0.20357

1’c···5 (form 2)-TS-**1’e···5** E = -1061.3496828 a.u., imaginary frequency -48.37cm^{-1}

O 1.58087 -3.45523 -0.13313
C 2.29940 -2.46616 -0.08267
N 1.71836 -1.19913 0.01571
C 2.33803 0.03939 0.07490
O 3.55485 0.19604 0.05879
N 1.44832 1.06290 0.12959
C 1.87113 2.40123 0.40913
O 2.01082 2.82327 1.53808
N 2.07023 3.12551 -0.70492
C 2.44353 4.52071 -0.67150
N 3.64513 -2.50911 -0.11750
C 4.36077 -3.75883 -0.19131
H 0.70426 -1.20269 0.05348
H 0.45258 0.83379 0.23556
H 1.85284 2.69159 -1.58204
H 4.12899 -1.62685 -0.05065
H 3.27214 4.66934 0.01798
H 1.61047 5.14902 -0.35240
H 2.75426 4.82411 -1.66783
H 4.09020 -4.31957 -1.08667

H 4.15872 -4.38826 0.67659
H 5.42561 -3.54277 -0.22477
C -3.60219 -1.38424 0.13518
C -3.35779 -0.01875 0.00097
C -4.44055 0.84339 -0.15982
C -5.74074 0.35511 -0.18486
C -5.97360 -1.00986 -0.05327
C -4.90002 -1.87927 0.10598
C -1.93586 0.52637 0.02948
O -1.01852 -0.31117 0.25456
O -1.77325 1.74503 -0.16601
H -2.75882 -2.04948 0.26267
H -4.24198 1.90190 -0.26140
H -6.57307 1.03763 -0.30716
H -6.98629 -1.39375 -0.07393
H -5.07512 -2.94334 0.20880

1’a···10 E = -1208.4301661 a.u.

O 2.57779 -3.16758 1.19929
C 2.39236 -2.15050 0.54608
N 1.13391 -1.70181 0.12477
C -0.00000 -2.49069 0.00000
O -0.00000 -3.70270 0.00000
N -1.13391 -1.70181 -0.12476
C -2.39237 -2.15049 -0.54608
O -2.57780 -3.16758 -1.19929
N -3.38855 -1.31183 -0.18290
C -4.73832 -1.53414 -0.62955
N 3.38854 -1.31184 0.18290
C 4.73832 -1.53415 0.62955
H 1.06269 -0.70271 -0.11554
H -1.06269 -0.70271 0.11554
H -3.20473 -0.46815 0.36700
H 3.20473 -0.46816 -0.36700
H -5.17458 -2.43161 -0.18397
H -4.78866 -1.64271 -1.71462
H -5.33826 -0.67542 -0.33464
H 5.17458 -2.43162 0.18396
H 4.78866 -1.64272 1.71462
H 5.33826 -0.67543 0.33464
C 2.19319 1.82572 -0.85578
N 1.07339 1.20311 -0.41382
C 0.00000 1.91582 -0.00000
C 0.00001 3.34027 0.00000
C 1.15460 3.99342 -0.48095
C 2.22644 3.27291 -0.91028
O 3.20179 1.14701 -1.20957
N -1.07338 1.20311 0.41382
C -2.19319 1.82572 0.85578
C -2.22643 3.27292 0.91028
C -1.15458 3.99342 0.48095
O -3.20178 1.14702 1.20957
H 1.17407 5.07989 -0.50297
H 3.12381 3.74911 -1.28445
H -3.12380 3.74912 1.28445
H -1.17405 5.07990 0.50298

1'c (form 1)+10 E = -1208.4229119 a.u.

O 2.22618 -2.82812 -0.65308
C 2.67665 -1.70409 -0.48700
N 1.84479 -0.67388 -0.03342
C 2.17515 0.66348 0.12446
O 3.31868 1.08922 0.02769
N 1.07624 1.44612 0.36235
C 1.12645 2.80240 0.73150
O 2.02968 3.29588 1.39151
N 0.03797 3.48446 0.32083
C -0.15871 4.85956 0.69640
N 3.96634 -1.38466 -0.73041
C 4.90438 -2.36799 -1.20713
H 0.88981 -0.97992 0.20257
H 0.14241 1.06926 0.10613
H -0.64894 3.05539 -0.30953
H 4.24546 -0.43718 -0.52917
H 0.57389 5.52302 0.23016
H -0.08468 4.98887 1.77717
H -1.15346 5.16077 0.37447
H 4.56508 -2.82105 -2.13958
H 5.05638 -3.16871 -0.48060
H 5.85738 -1.87543 -1.38701
C -1.33610 -2.66136 1.19472
N -1.01156 -1.43578 0.69103
C -1.93084 -0.70369 0.02515
C -3.25049 -1.17759 -0.23218
C -3.58451 -2.46319 0.25286
C -2.67065 -3.19055 0.94566
O -0.51255 -3.32714 1.86652
N -1.53491 0.52602 -0.38798
C -2.37988 1.35468 -1.04560
C -3.71719 0.90278 -1.35475
C -4.12390 -0.33264 -0.94470
O -1.99836 2.51919 -1.37614
H -4.58211 -2.85408 0.06866
H -2.90083 -4.17315 1.33784
H -4.37202 1.57299 -1.89698
H -5.13162 -0.67980 -1.15909

1'c (form 2)+10 E = -1208.4270528 a.u.

O 3.90208 -2.24144 0.10809
C 3.79113 -1.03053 0.25037
N 2.60398 -0.39157 -0.11728
C 2.31650 0.96174 -0.03327
O 3.13271 1.80045 0.32502
N 1.01873 1.24632 -0.37151
C 0.51856 2.55029 -0.56705
O 1.21027 3.48815 -0.93860
N -0.80496 2.63781 -0.34044
C -1.52246 3.85631 -0.61356
N 4.77375 -0.25580 0.75855
C 6.04272 -0.81125 1.15226
H 1.89905 -1.03397 -0.51757
H 0.33055 0.47225 -0.39603
H -1.35936 1.83065 -0.02642

H 4.59533 0.73618 0.78768
H -1.50595 4.10743 -1.67711
H -1.10011 4.69882 -0.06402
H -2.55327 3.71096 -0.29938
H 6.54816 -1.29556 0.31450
H 5.92736 -1.54946 1.94751
H 6.67268 -0.00380 1.51837
C -0.46121 -2.09217 -0.82129
N -0.94731 -0.89809 -0.40636
C -2.22933 -0.77329 0.01804
C -3.11163 -1.89176 0.05574
C -2.60813 -3.13840 -0.36997
C -1.32194 -3.25537 -0.80227
O 0.73851 -2.18567 -1.21592
N -2.62103 0.46286 0.39547
C -3.88932 0.69688 0.83052
C -4.81970 -0.41962 0.89912
C -4.42766 -1.66476 0.51732
O -4.25435 1.85304 1.16471
H -3.26304 -4.00572 -0.34740
H -0.90822 -4.20032 -1.12916
H -5.82306 -0.22190 1.25630
H -5.12123 -2.50092 0.56134

1’e···10 E = -1208.4141332 a.u.

O 0.82842 3.28326 -0.80349
C 1.66870 2.41225 -0.63693
N 1.27940 1.14450 -0.19037
C 2.06859 0.04005 0.00130
O 3.30343 0.06791 -0.01863
N 1.33837 -1.09993 0.22017
C 1.80395 -2.35008 0.64268
O 1.01250 -3.26202 0.82588
N 3.13487 -2.47293 0.84568
C 3.71459 -3.72276 1.26605
N 2.98548 2.59629 -0.88224
C 3.49450 3.86186 -1.34532
H 0.28126 1.07681 0.10547
H 0.33086 -1.08798 -0.04730
H 3.71027 -1.68775 0.58929
H 3.60416 1.83974 -0.64081
H 3.58783 -4.50343 0.51312
H 3.26065 -4.07399 2.19277
H 4.77822 -3.57094 1.43536
H 3.37332 4.64848 -0.59770
H 2.98483 4.18051 -2.25451
H 4.55427 3.74828 -1.56287
C -2.03279 1.76478 1.53364
N -1.40931 0.84174 0.75102
C -2.11476 -0.04758 0.01434
C -3.53918 -0.04222 -0.01138
C -4.19886 0.92093 0.78224
C -3.48687 1.79965 1.53971
O -1.36812 2.56973 2.23415
N -1.38727 -0.94103 -0.69531
C -1.98899 -1.86613 -1.49336
C -3.44230 -1.88606 -1.55712
C -4.17598 -0.99973 -0.82985
O -1.30712 -2.68510 -2.16080
H -5.28599 0.94840 0.78055

H -3.97358 2.54494 2.15651
H -3.91089 -2.62703 -2.19294
H -5.26251 -1.01639 -0.87238

1’a···10-TS-**1’c···10 (form 1)** (TS1, Figure 10 in main text)
E = -1208.4117724 a.u., imaginary frequency -51.54 cm^{-1}

O -3.20677 -0.96520 1.75490
C -3.07039 -0.49076 0.64234
N -1.86884 0.13360 0.20833
C -1.73432 1.49288 0.23641
O -2.65871 2.24857 0.49285
N -0.43461 1.89790 -0.03240
C -0.04434 3.19317 -0.38138
O -0.80803 4.05330 -0.80076
N 1.28683 3.39012 -0.24480
C 1.89498 4.62209 -0.67220
N -4.02053 -0.53737 -0.30929
C -5.30772 -1.15312 -0.09926
H -1.06965 -0.50005 -0.00313
H 0.31276 1.19187 0.03923
H 1.90111 2.66429 0.13809
H -3.79153 -0.15861 -1.20777
H 1.50487 5.48122 -0.12179
H 1.73258 4.80819 -1.73677
H 2.96585 4.55100 -0.49199
H -5.80275 -0.72313 0.77128
H -5.21108 -2.22878 0.05550
H -5.92585 -0.97798 -0.97616
C -0.09822 -2.96408 -0.68728
N 0.25862 -1.68277 -0.39618
C 1.52894 -1.36504 -0.06379
C 2.55295 -2.35206 0.02301
C 2.19296 -3.68974 -0.25678
C 0.91596 -4.00365 -0.60333
O -1.27779 -3.24246 -1.01900
N 1.77859 -0.05638 0.18304
C 3.02192 0.36331 0.52037
C 4.09285 -0.60406 0.62973
C 3.84587 -1.92114 0.38310
O 3.24420 1.59286 0.73382
H 2.95398 -4.46344 -0.19020
H 0.61720 -5.02150 -0.82235
H 5.07700 -0.24619 0.90434
H 4.64323 -2.65601 0.46077

1’c···10 (form 1)-TS-**1’c···10 (form 2)** (TS2, Figure 10 in main text)
E = -1208.4169602 a.u., imaginary frequency -83.41 cm^{-1}

O 3.08479 -2.30784 -0.73240
C 3.24917 -1.12403 -0.48002
N 2.18762 -0.35632 0.01255
C 2.13967 1.01857 0.16917
O 3.10892 1.74964 0.03363
N 0.87272 1.45466 0.46401
C 0.50783 2.80085 0.67879
O 1.26050 3.63847 1.14989
N -0.77084 3.03884 0.33480

C -1.38259 4.32336 0.54984
N 4.42751 -0.48526 -0.64634
C 5.56799 -1.15665 -1.21539
H 1.33268 -0.88233 0.20288
H 0.11231 0.78330 0.35137
H -1.32225 2.30256 -0.10102
H 4.43605 0.50544 -0.45965
H -0.85154 5.11490 0.01772
H -1.40300 4.58602 1.60964
H -2.40127 4.26870 0.17535
H 5.34643 -1.55500 -2.20682
H 5.89813 -1.98456 -0.58657
H 6.38129 -0.43997 -1.30152
C -0.54887 -2.53061 1.07998
N -0.72003 -1.28432 0.55345
C -1.90050 -0.92356 -0.00337
C -2.99339 -1.83256 -0.11699
C -2.81206 -3.13740 0.39294
C -1.63693 -3.49088 0.97781
O 0.53000 -2.84283 1.64060
N -1.99411 0.35469 -0.43660
C -3.14195 0.82877 -0.98820
C -4.26568 -0.07167 -1.15801
C -4.17666 -1.35876 -0.72080
O -3.22110 2.04103 -1.33206
H -3.62842 -3.85156 0.31579
H -1.47582 -4.48230 1.38277
H -5.16643 0.31572 -1.61758
H -5.01951 -2.03725 -0.82738

Additional discussion

Opposite effect of substituent on association

When two extreme conformers are taken into account (**1a** and **1e**) the said *opposite effect* is caused by the change of conformation in **1**. The interaction of conformers with variable electron density (substituent) at the oxygen atoms in benzoates is various depending on rotational state. In complexes of benzoates with **1a** the substituent effect should be classical making interaction stronger as substituent became more electron donating. On the other hand in benzoates associated to **1e** the more electron donating substituent strengthens the NH···O hydrogen bonds but at the same time the repulsion between O8/O10 (in **1**) and negatively charged oxygen atoms in benzoates became stronger (Figure 3). While the change from **1e** to **1a** leads by other conformations the whole range of conformations and interaction possibilities influences averaged chemical shift causing sigmoidal shape of the titration curve.

The above discussion on nonlinearity of association leads to the conclusion known from the other publications that preorganization [2] of organic molecules and their rigidness is crucial in the light of supramolecular chemistry, while flexibility causes problems in predicting properties. However, it is worth mentioning that in biochemical reactions based on enzymes the said flexibility allows effective interactions due to the geometric demands of molecules (also in metal-organic frameworks [2]) and thus it is crucial for reactions responsible for such processes.

*Changes in conjugation of **1'** upon conformational changes*

The electron conjugation in some dibuthyltriuret forms stabilized by intramolecular hydrogen bonding additionally stabilizes these conformations. Such stabilization is called resonance-assisted hydrogen bond [3] and is expressed by strengthening of intramolecular hydrogen bonding that, in turn, influences the overall stability. Here two approaches were used to test geometry and associated energy changes: *a*) the HOMA index [4] based on geometry (bonds lengths) was used to describe the changes in electron delocalization/bond length averaging upon association and *b*) the single point calculations on *extracted* geometries of **1'** taken form optimization of its structure in the complex. These were run to find what is the relative energy of respective **1'a** form in complexes with **5** and with **10**. This gave us the energy of geometry perturbation of **1'a** upon association ($E_{(\mathbf{1'a})}^{rel}$). The $E_{(\mathbf{1'a})}^{rel}$ was calculated for the **1'a** form since it is not stabilized by intramolecular hydrogen bond(s), thus the changes should be the highest among all rotameric forms. Table S2 collects the detailed data.

Table S2. The HOMA[a] index values for conformers and relative energy of **1'a** in associates $E_{(\mathbf{1'a})}^{rel}$ [kJ/mol]

Form	HOMA	$E_{(\mathbf{1'a})}^{rel}$
**1'a**	0.599	0.0
**1'b**	0.685	-
**1'c**	0.654	-
**1'd**	0.732	-
**1'e**	0.702	-
**1'a** in **1’a···5** complex[b]	0.685	13.8
**1'a** in **1’a···10** complex	0.721	11.6

[a] - all C=O and C-N bonds taken into account,
[b] - HOMA for anions is practically constant (+/- 0.02)

The $E_{(\mathbf{1'a})}^{rel}$ refers to the energy gain by **1'a** caused by distortion from the optimal geometry upon association. It is worth noting that association of **1'a** form causes this form to increase its energy. On the other hand the conjugation expressed by HOMA is

higher due to the planarization tendency and partial bond length equalization in associated **1'a**. This suggests that the energy gain is caused by electron repulsion between oxygen atoms (**1'a** in **1'a···5** is flat and bent, while **1'a** in **1'a···10** is twisted along its longitudinal axis). The planarization of **1'a** in complex with **5** is caused by its tendency of acting as pincer for the benzoate anion (**5**), while, due to the size of naphthyridine dianion, it is not possible to act the same way for **10**. The higher HOMA index in **1'a** in the complex express better conjugation in associated form telling that association is worth the $E_{(\mathbf{1'a})}^{rel}$ to be paid.

Discussion of the substituent effect on association

As it was shown before the substituent effect influences the association of benzoates [5, 6]. However, the interaction with benzoates also depends on the structure of counterpart carrying hydrogen bond donors. In the current study the substituent effect is not strong and does not have a highly linear character. Nevertheless, the general substituent effect is able to deliver additional information on the structure of the counterpart. The highest association constant among the series of benzoates was obtained for the unsubstituted derivative. This suggests that any subtle change in electron density at the oxygen atoms of the carboxylate causes the nonlinearity of association constants with the character of the substituent. On the other hand the CIS values for H1/H7 and H3/H5 correlate with the Hammett substituent constant giving values R = 0.890 (0.94 when CF_3 is removed from analysis) and R = 0.80, respectively and no correlation of the CIS values for CH_2 group. These observations may be explained by *a*) multiple equilibria associated with the rotamerism in **1** that lead to the presence of various forms in solution, *b*) the fact that the said forms and their interaction depends not only on the rotameric state but also on intramolecular electronic repulsion, intramolecular hydrogen bonding,

intermolecular secondary interactions and finally *c*) the association depends in various ways upon fluctuations of the electron density. The mentioned fluctuations of electron density are dependent from the substituent. Also the interaction of the substituent's electron lone pair(s) with **1** may play a role in association. The fact that CIS values for NH protons correlate with the substituent constant but this is not realized for the CH_2 group supports the conclusion of the non-regularity of association changes in complexes. This shows parallels to the opposite effect of the substituent on the basicity of C=O oxygen and acidity of OH group in substituted acids and their complexes.[6]

Computations

In all calculations the methyl group was used instead of *n*-Bu. The structure is labeled as **1'**. For all forms of **1'** and its complexes full geometry optimizations were performed. Moreover we have also run the calculations that helped us to understand the rotamerism in the studied complexes (rotational transition states in **1'** and its complex with **5** and **10**). Also, the various doubly hydrogen-bonded complexes of **1'** were examined.

The rotamerism in 1' and its interaction with ***5***

Table S3 collects the relative energy of rotamers (Figure 2 in the main text) of **1'** and the QTAIM-derived [7] data of electron density [ρ] at H-BCP (hydrogen bond critical point), its Laplacian [$\nabla^2\rho$], energy of hydrogen bond [E_{HB}, kJ/mol] obtained by Espinosa [8] approach and its lengths [HB_{dis}, Å]. The positive values of Laplacian tells that the interaction is of hydrogen bond nature.[7, 9]

Table S3. Relative energy and QTAIM data for intramolecular hydrogen bonds in rotamers of **1'**

Form	Relative energy [kJ/mol]	Atoms<br>E_{HB}<br>HB_{dis}	 ρ<br>$\nabla^2\rho$	Atoms<br>E_{HB}<br>HB_{dis}	 ρ<br>$\nabla^2\rho$
**1'a**	61.9	–	–	–	––
**1'b**	25.4	H5⋯O8		–	–
		-38.3	0.035		
		1.838	0.123		
**1'c**	31.2	H1⋯O9		––	–
		-28.6	0.027		
		−−1.9<br>46	0.104		
**1'd**	0.0	H5⋯O8		H7⋯O9	
		−32.7	0.031	–28.4	0.027
		1.895	0.113	1.949	0.104
**1'e**	0.4	H1⋯O9		–	–
		−27.9	0.027		
		1.954	0.103		

The data in Table S3 are in agreement with experimental results[10] and general observations known as Etter's rules[11] regarding formation of intramolecular

hydrogen bonds, i.e. two, the most stable forms are **1'd** and **1'e** - both stabilized by two intramolecular hydrogen bonds (the sum of E_{HB} form these to are -61.1 and -55.8 kJ/mol, respectively). The relative energy between these forms is small, but to convert **1'd** into **1'e** it is necessary to pass through structure **1'c** - rotations about N3-C4 (**1'd→1'c**) and C2-N3 (**1'c→1'e**). This path (**dce**) yields with two rotational transition states. Forms **1'd** and **1'e** are capable to form complexes with benzoate anion by double hydrogen-bonding, while **1'c** can form two various hydrogen bonded complexes stabilized by hydrogen bonds and attractive secondary interaction (Figure S32).

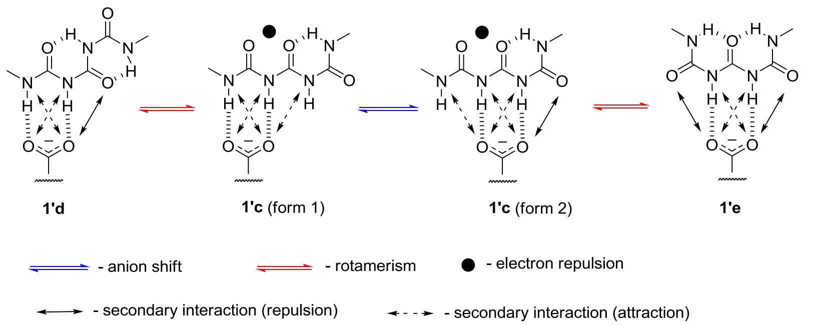

Figure S32. The possible complexes of **1'c–e** with the carboxylic acid anion (secondary interactions shown).

The strongest intramolecular electronic repulsion on one hand and the strongest intermolecular interaction on the other (up to four hydrogen bonds) should be present in associate with **1'a** (see Figure 3 in the main text, left structure). If so, the alternate rotameric path should be considered starting form the same, the most stable, **1'd** form. This path (**dba**) starts with rotation about N5–C6 bond (**1'd→1'b**) and then N3–C4 (**1'b→1'a**). On the other hand **1'e** form may be converted to **1a** in two steps, *a*) rotation about C2–N3 (**1'e→1'c**) and *b*) rotation about N5–C6 (**1'c→1'a**). In Figure S33 three paths (the third with the **1'c-TS-1'a** transition state) are shown together with differences in energies describing rotamerism in **1'**.

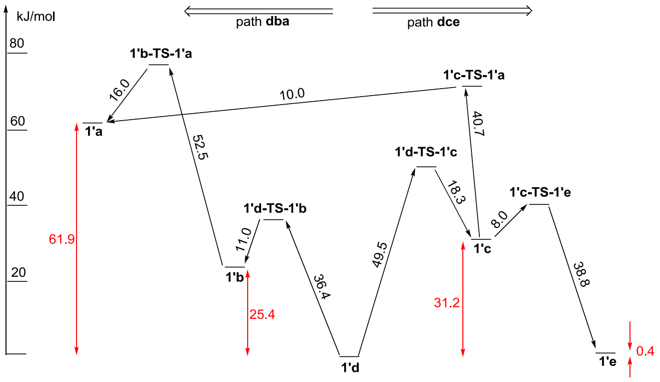

Figure S33. The energetic of rotamerism in **1'**

The **dce** path starting from the lowest in energy **1'd** is more probable. However, it is worth noting that it must pass through the **1'c** form. If **1'c** form is probable the form **1'b** should possibly exist (the **1'c** relative energy is 31.2 while **1'd-TS-1'b** is 36.4 kJ/mol, respectively). It is important that **1'c-TS-1'a** and **1'a** are quite close in energy but the **1'c-TS-1'a** energy is quite high with respect to **1'd** (71.9 kJ/mol). The relatively high energy of **1'a** with respect to **1'd** is caused by electronic repulsion between oxygen atoms (Figure 2) making this form not flat but twisted (see Figure S29, *side view*). In Figure S34 similar calculations are presented but the rotamerism is of "anion-assisted" type. The anion-assisted rotamerism passing through the **1'c-TS-1'a** state was omitted due to much lower in energy **1'd-TS-1'c** and **1'c-TS-1'e** states.

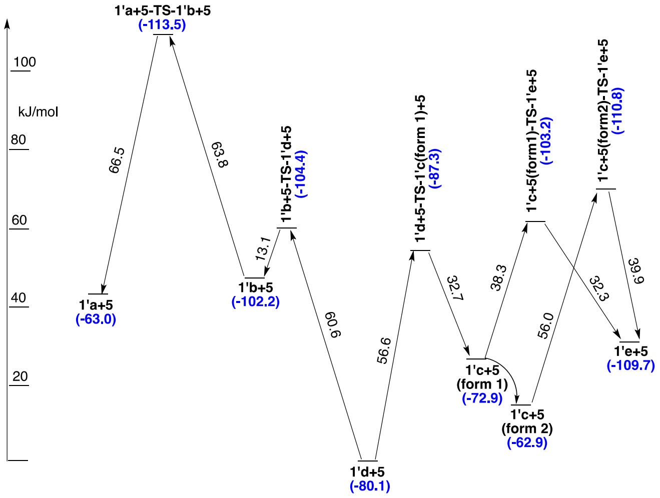

Figure S34. The relative energy of rotamers in **1'···5** complex (interaction energy (E_{int}) in blue [kJ/mol])

The energy of transition states in associates show that in most cases the transition state energy (versus the most stable **1’d···5** complex) is higher than the respective transition state energy in uncomplexed **1'**. However, when one compares Figure S33 and S34 side-by-side it is easy to note that for **1’a···5** and **1’c···5** their relative energy is lower than **1'a** and **1'c** with respect the most stable form (**1’d···5** and **1'd**, respectively). This is observed for complexes that are able to form more than two intermolecular hydrogen bonds suggesting that these rotamers are more probable to exist as associates than in **1'** if only the rotational barrier is reached. Moreover, since the repulsive inter- (secondary) or intramolecular (electronic repulsion) interactions exist in every associate the intermolecular interactions in transition states are stronger than that in structures in energy minima. This is caused by twisting groups responsible for repulsion by ca. 90 degrees in the transition state. The subtle balance between these forces is important in such a complicated equilibrium independently from the relatively small size of molecules.

Based on the QTAIM it is possible to calculate the hydrogen bond energy also for weak interactions of CH···X type [12] or relatively long hydrogen bonds.[6] Table S4 collects the QTAIM derived data for complexes of **1’···5**.

Table S4. The E_{HB} [kJ/mol] values[a] for hydrogen bonds in complexes of **1’···5**

Complex	H1	H3	H5	H7	$\Sigma E_{HB}^{(inter)}$	$\Sigma E_{HB}^{(intra)}$
**1’a···5**	-13.7	-34.9	-34.9	-13.7	-97.2	-
**1’b···5**	-37.1	-35.8	*-46.0*	-	-72.9	*-46.0*
**1’c···5** (form 1)	-22.7	-30.8	-32.8	*-33.0*	-86.3	*-33.0*
**1’c···5** (form 2)	-31.6	-38.9	-18.6	*-31.5*	-89.2	*-31.5*
**1’d···5**	-36.7	-37.9	*-38.0*	*-31.8*	-74.7	*-69.7*
**1’e···5**	*-34.4*	-39.6	-39.6	*-34.4*	-79.3	*-68.7*

[a] intramolecular hydrogen bond in *italic*

Taking into account the BSSE and ZPE corrected energy of the **1’d···5** complex has the lowest energy. On the other hand the energy of intermolecular interaction is the highest for **1’e···5**. This value is, however, comparable to other values including stabilization of the transition states E_{int} (Figure S34). The said numbers confirm **1** is a flexible molecule with the ease of association, rotamerism and thus formation of multiple supramolecular structures.

*Interaction of **1'** with **10***

The interaction of **1** with **10** showed that two forms coexist in solution. The following computational data with a proposed mechanism leading to a coexistence of two forms support experimental findings. In Figure S35 the energy diagram shows the relative energy of transition states and locally stable form (**1’c···10** (form1)) between more stable forms (in red, Figure 10 in the main text). Table S5 collects the hydrogen bond energies for the most probable **1’···10** complexes and transition states referring to those.

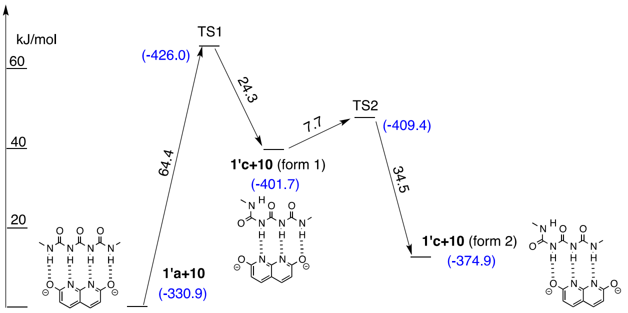

Figure S35. The energy diagram for **1’···10** complexes (E_{int} in blue [kJ/mol])

Table S5. The E_{HB} [kJ/mol] values for hydrogen bonds in complexes of **1'** with **10**

Complex	H1[a]	H3	H5	H7	$\Sigma E_{HB}^{(inter)}$	ΣE_{HB} (intra)
**1’a···10**	-35.7	-28.7	-28.7	-35.7	-128.8	-
**TS1**		-38.7	-28.6	-36.0	-103.3	-
**1’c···10** (form 1)	-35.9	-21.3	-38.6	-37.7	-97.6	-35.9
**TS2**[b]	-34.4	-16.2	-12.7	-18.1	-71.0	-34.4
			-11.3	-12.7		
**1’c···10** (form 2)	-36.2	-39.4	-33.7	-30.2	-103.4	-36.2

[a] hydrogen bonds order as in Figure 15 from left to right side, [b] - H5 and H7 protons form bifurcated hydrogen bonds in the transition state (**TS2**)

The **1’e···10** complex was not taken into account because its energy relative to **1’a···10** is 92.2 kJ/mol so it is twice higher than that for **1’c···10** (form 1) and ca. 30 kJ/mol higher than the most energetic transition state. The close in energy **1’a···10** and **1’c···10** (form 2) forms are those that have been proposed after inspection of the VT ^{1}H NMR spectra. The number of nonequivalent protons in the said spectra (Figure 9 in the main text) is in agreement with the proposed structures showing the **1’a···10** complex exists at room temperature while it converts to **1’c···10** (form 2) at lower temperatures. Moreover, the GIAO[13] calculated data (same method and basis set as for optimization) for complex **1’c···10** (form 2) show the difference in shielding of the H3 vs H6 proton to be 0.2 ppm, while between H4 and H5 0.05 ppm. That is very close to the observer values (Figure 9 in the main text, lowered temperature).

The changes of the geometry in the light of the conjugation, based on HOMA index of aromaticity and single point energy calculations was described in above.

Mass spectrometry

Samples preparation: Analytical solutions of **1** with benzoate and naphthyridine anions (1:1) and of only **1** were prepared in chloroform and diluted in methanol to give a final concentration of 10μM of dibuthyltriuret and of 10μM of proper salt **5**, **10**.

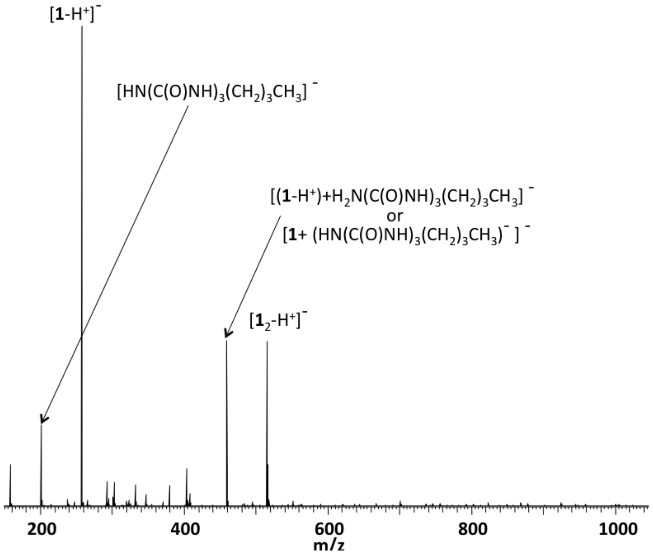

Figure S36**.** ESI(-) HRMS mass spectrum of **1**···**5** mixture in chloroform/methanol solution

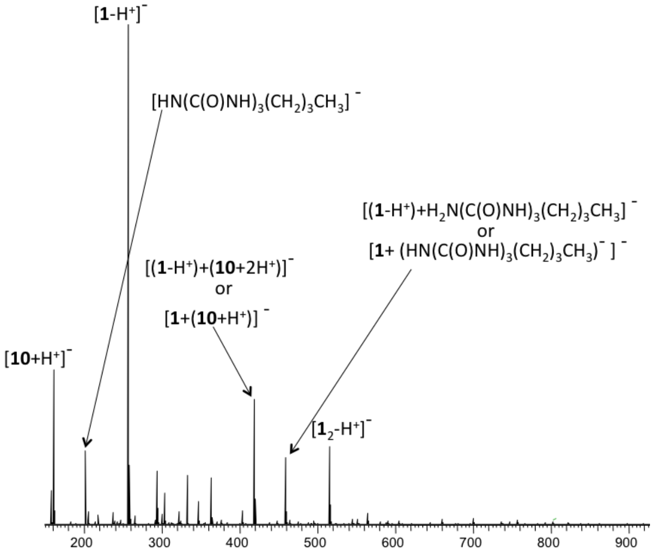

Figure S37. ESI(-) HRMS mass spectrum of **1**···**10** mixture in chloroform/methanol solution

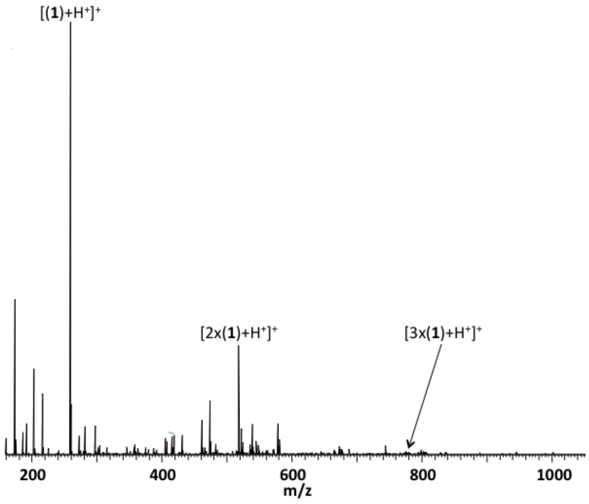

Figure S38. ESI(+) HRMS mass spectrum of **1** in chloroform/methanol solution

References

[1] K. A. Connors, *Binding Constants: The Measurement of Molecular Complex Stability*, John Wiley and Sons, New York, **1987**.

[2] J. B. Wittenberg, L. Isaacs, in *Supramolecular Chemistry*, John Wiley & Sons, Ltd, **2012**.

[3] P. Gilli, V. Bertolasi, V. Ferretti, G. Gilli, *J. Am. Chem. Soc.* **1994**, *116*, 909-915. doi: 10.1021/ja00082a011; G. Gilli, F. Bellucci, V. Ferretti, V. Bertolasi, *J. Am. Chem. Soc.* **1989**, *111*, 1023-1028. doi: 10.1021/ja00185a035

[4] J. Kruszewski, T. M. Krygowski, *Tetrahedron Lett.* **1972**, *13*, 3839-3842. doi: 10.1016/S0040-4039(01)94175-9

[5] B. Ośmiałowski, K. Mroczyńska, E. Kolehmainen, M. Kowalska, A. Valkonen, M. Pietrzak, K. Rissanen, *J. Org. Chem.* **2013**, *78*, 7582-7593. doi: 10.1021/jo4011393

[6] B. Ośmiałowski, *J. Mol. Model.* **2014**, *20*, 2356-2366. doi: 10.1007/s00894-014-2356-8

[7] R. F. W. Bader, *Atoms in Molecules: A Quantum Theory*, Oxford University Press, New York, **1990**.

[8] E. Espinosa, I. Alkorta, J. Elguero, E. Molins, *J. Chem. Phys.* **2002**, *117*, 5529-5542. doi: 10.1063/1.1501133; E. Espinosa, I. Alkorta, I. Rozas, J. Elguero, E. Molins, *Chem. Phys. Lett.* **2001**, *336*, 457-461. doi: 10.1016/S0009-2614(01)00178-6; E. Espinosa, C. Lecomte, E. Molins, *Chem. Phys. Lett.* **1999**, *300*, 745-748. doi: 10.1016/S0009-2614(98)01399-2; E. Espinosa, E. Molins, *J. Chem. Phys.* **2000**, *113*, 5686-5694. doi: 10.1063/1.1290612; E. Espinosa, E. Molins, C. Lecomte, *Chem. Phys. Lett.* **1998**, *285*, 170-173. doi: 10.1016/S0009-2614(98)00036-0; E. Espinosa, M. Souhassou, H. Lachekar, C. Lecomte, *Acta Cryst.* **1999**, *B55*, 563-572. doi: 10.1107/S0108768199002128

[9] L. A. Popelier, *Atoms in molecule. An introduction.*, Pearson Education, London, **2000**.

[10] D. Carlstrom, H. Ringertz, *Acta Cryst.* **1965**, *18*, 307-313. doi: 10.1107/S0365110X65000737

[11] M. C. Etter, *Acc. Chem. Res.* **1990**, *23*, 120-126. doi: 10.1021/ar00172a005

[12] A. Allerhand, P. Von Rague Schleyer, *J. Am. Chem. Soc.* **1963**, *85*, 1715-1723. doi: 10.1021/ja00895a002; B. Ośmiałowski, E. Kolehmainen, A. Valkonen, M. Kowalska, S. Ikonen, *Struct. Chem.* **2013**, *24*, 2203-2209. doi: 10.1007/s11224-013-0283-4; E. J. Corey, J. J. Rohde, A. Fischer, M. D. Azimioara, *Tetrahedron Lett.* **1997**, *38*, 33-36. doi: 10.1016/S0040-4039(96)02248-4

[13] K. Wolinski, J. F. Hinton, P. Pulay, *J. Am. Chem. Soc.* **1990**, *112*, 8251-8260. doi: 10.1021/ja00179a005
